# Comprehensive functional analysis of the *tousled-like kinase 2* frequently amplified in aggressive luminal breast cancers

**DOI:** 10.1038/ncomms12991

**Published:** 2016-10-03

**Authors:** Jin-Ah Kim, Ying Tan, Xian Wang, Xixi Cao, Jamunarani Veeraraghavan, Yulong Liang, Dean P. Edwards, Shixia Huang, Xuewen Pan, Kaiyi Li, Rachel Schiff, Xiao-Song Wang

**Affiliations:** 1 Lester & Sue Smith Breast Center, Baylor College of Medicine, Houston, Texas 77030, USA; 2 Dan L. Duncan Cancer Center, Baylor College of Medicine, Houston, Texas 77030, USA; 3 Department of Medicine, Baylor College of Medicine, Houston, Texas 77030, USA; 4 University of Pittsburgh Cancer Institute, University of Pittsburgh, Pittsburgh, Pennsylvania 15213, USA; 5 Department of Pathology, University of Pittsburgh, Pittsburgh, Pennsylvania 15213, USA; 6 Department of Surgery, Baylor College of Medicine, Houston, Texas 77030, USA; 7 Department of Pathology & Immunology, Baylor College of Medicine, Houston, Texas 77030, USA; 8 Department of Molecular and Cellular Biology, Baylor College of Medicine, Houston, Texas 77030, USA; 9 Department of Biochemistry and Molecular Biology, Baylor College of Medicine, Houston, Texas 77030, USA

## Abstract

More aggressive and therapy-resistant oestrogen receptor (ER)-positive breast cancers remain a great clinical challenge. Here our integrative genomic analysis identifies *tousled-like kinase 2* (*TLK2*) as a candidate kinase target frequently amplified in ∼10.5% of ER-positive breast tumours. The resulting overexpression of *TLK2* is more significant in aggressive and advanced tumours, and correlates with worse clinical outcome regardless of endocrine therapy. Ectopic expression of TLK2 leads to enhanced aggressiveness in breast cancer cells, which may involve the EGFR/SRC/FAK signalling. Conversely, TLK2 inhibition selectively inhibits the growth of *TLK2*-high breast cancer cells, downregulates ERα, BCL2 and SKP2, impairs G1/S cell cycle progression, induces apoptosis and significantly improves progression-free survival *in vivo*. We identify two potential TLK2 inhibitors that could serve as backbones for future drug development. Together, amplification of the cell cycle kinase *TLK2* presents an attractive genomic target for aggressive ER-positive breast cancers.

## Abstract (web summary)

Luminal B oestrogen receptor positive breast cancers are generally aggressive tumors with poor outcomes. Here, the authors show that the kinase TLK2 is amplified and overexpressed in these tumors and correlates with reduced survival, TLK2 inhibition induces apoptosis *in vitro* and improves survival in mice.

## Graphical abstract

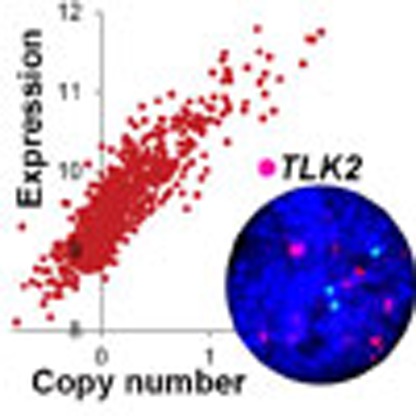

A vast majority of breast cancers express the oestrogen receptor (ER+) and can be treated with endocrine therapy; however, the clinical outcome varies radically between different patients. ER+ breast cancers are also known as luminal breast cancers and can be subdivided into A and B subtypes. The luminal B tumours are more aggressive ER+ breast cancers characterized by poorer tumour grade, larger tumour size and higher proliferation index. Clinically, such tumours are prone to develop endocrine resistance, which poses a great challenge to clinical management. Identifying the genetic aberrations underlying the enhanced aggressiveness of these tumours, and developing effective therapeutic strategies to target them, are in high demand. Recent prominent success of the CDK4/6-specific inhibitors in clinical trials for advanced breast cancers have attracted wide-spread attention to the potential of cell cycle kinases as viable drug targets in breast cancer^1^. Thus, discovering new cell cycle kinase targets that can tackle the more aggressive ER+ breast cancers will be of critical clinical significance.

Genomic amplifications lead to deregulations of oncogenes to which cancer cells become often addicted in specific tumours. Such events, however, usually affect a large number of genes in cancer genomes, which make it difficult to identify the primary oncogene targets of these amplifications. In our previous study, we discovered that cancer genes possess distinctive yet complicated ‘gene concept signature', which include cancer-related signalling pathways, molecular interactions, transcriptional motifs, protein domains and gene ontologies^2^. Based on this observation, we developed a Concept Signature (or ConSig) analysis that prioritizes the biological importance of candidate genes underlying cancer via computing their strength of association with those cancer-related signature concepts (http://consig.cagenome.org)^2^,^3^,^4^. In our previous study, we have applied this analysis to reveal the primary target genes of chromosome 17q amplifications in breast cancer^5^. Here we postulate that the ConSig analysis may be used to effectively nominate dominantly acting cancer genes from the genomic amplifications in cancer at a genome-wide scale, which can be further translated into viable therapeutic targets by interrogating pharmacological databases (Fig. 1a). Toward this end, we have assembled a genome-wide analysis called ‘ConSig-Amp' to discover viable therapeutic targets in cancer from multi-dimensional genomic data sets.

Applying this analysis to the genomic data from The Cancer Genome Atlas (TCGA) nominated a new oncogene target called *tousled-like kinase 2* (*TLK2*) frequently amplified in aggressive luminal breast cancer. Tousled-like kinases (TLKs) are nuclear serine/threonine kinases that promote chromatin assembly during S-phase and also chromosome segregation during mitosis^6^,^7^,^8^. The *TLK* gene family includes two members, *TLK1* and *TLK2* (Supplementary Fig. 1a)^9^. Most, if not all, of the reports about the function of TLKs focus on the study of TLK1, while the function of TLK2 and its role in human cancers are still largely unknown. To date, there is no functional characterization of TLK2 in breast cancer, although TLK2 single nucleotide polymorphism has been associated with increased breast cancer risk^10^, and most recently TLK2 has been reported as an amplicon-associated highly phosphorylated kinase in luminal breast cancer^11^. Here we discovered that TLK2 overexpression endows enhanced invasiveness of luminal breast cancers, and appears to be addictive for *TLK2*-amplified breast cancers so that TLK2 inhibition renders decreased cancer cell viability and increased apoptosis. This suggests that TLK2 may serve as an attractive genomic target for the aggressive luminal breast cancers harbouring *TLK2* amplifications.

## Results

### *TLK2* as a lead target amplified in ER+ breast cancers

**Figure 1:**
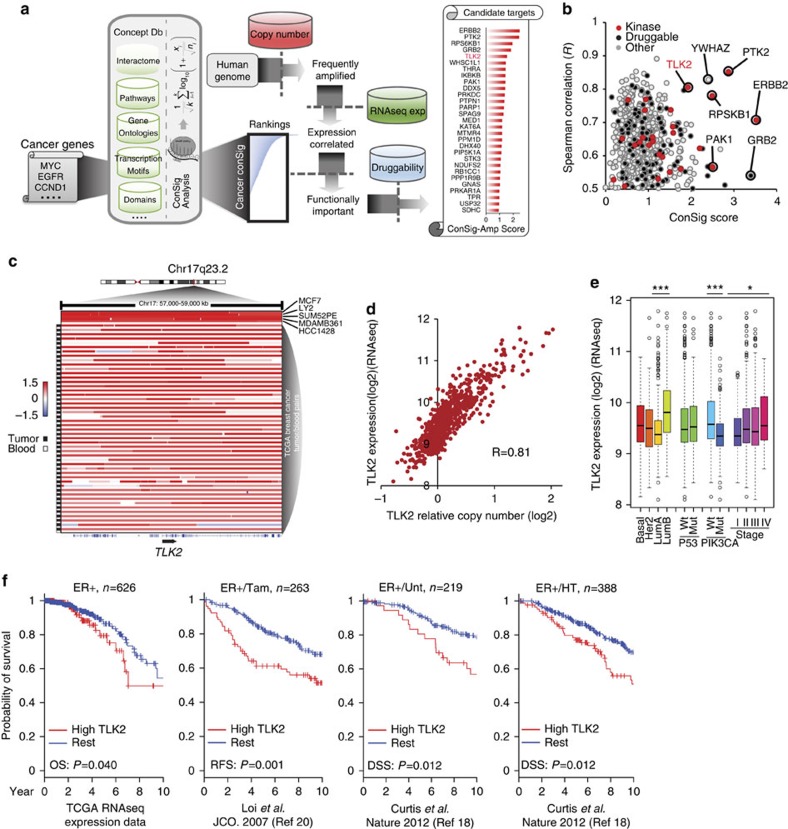
ConSig-Amp identifies *TLK2* as a candidate druggable target frequently amplified in breast cancer. (**a**) The bioinformatics workflow of ConSig-Amp to discover therapeutically relevant oncogene targets in cancer at genome-wide scale based on copy-number and RNAseq data sets. The ConSig-Amp score is calculated by multiplying the ConSig score (see Methods) with the correlation between gene expression and copy number. (**b**) Prioritizing amplified breast cancer oncogene targets by ConSig score and Spearman's correlation between copy number (Affymetrix SNP 6.0 array) and gene expression (RNAseq). Data shown here are from TCGA. (**c**) Representative copy-number data showing amplifications at the *TLK2* locus in paired breast tumour and peripheral blood (data from TCGA^52^), or breast cancer cell lines (data from Heiser *et al*.^21^). This figure is based on Affymetrix SNP 6.0 array data annotated with genome build hg18. Positive cell line or tumour samples are sorted based on the level of *TLK2* amplifications, and the structures of genes involved in the presented region are shown under the illustration. (**d**) *TLK2* expression (based on RNAseq data) is primarily regulated by gene copy number (based on Affymetrix SNP 6.0 array data). The Spearman's correlation is *R*=0.81. (**e**) *TLK2* expression in different breast cancer subtypes based on RNAseq data. Copy number and RNAseq expression data shown in **d**,**e** are from TCGA. The whiskers indicate the max and min values (excluding outliers) and horizontal lines represent the 1st, 2nd and 3rd quartiles. *P<0.05; ^***^P<0.001. (**f**) Kaplan–Meier plots based on multiple gene expression data sets showing correlation of *TLK2* overexpression with the outcome of systemically untreated or endocrine-treated ER+ breast cancer patients. HT, hormone treated; Tam, tamoxifen-treated; Unt, untreated. *P* values are calculated based on log-rank tests.

To systematically reveal new therapeutic targets, we applied the ‘ConSig-Amp' analysis to the genomic data sets for breast cancers from TCGA^12^. First, we identified all human genes that are amplified in >5% of ER+ breast cancers based on copy-number data. Candidates were then benchmarked with the ConSig-Amp score which is calculated by multiplying ConSig score (Methods) by the correlation between gene expression and copy number, to prioritize biologically important targets that are upregulated by genomic amplifications. Potentially druggable candidates were then selected according to a drug-target database compiled from multiple sources^13^,^14^,^15^. This revealed several known kinase targets in breast cancer such as *ERBB2, PAK1, RPS6KB1* and *PTK2* (refs 16, 17), together with a new candidate kinase target, *TLK2* (Fig. 1b; Supplementary Table 1).

Copy-number data from TCGA show that *TLK2* is amplified in about ∼9% of all breast cancers (Fig. 1c), and such events are more frequent in ER-positive than negative breast cancers (10.5 versus 2.9%) (Supplementary Fig. 1b). *TLK2* locates in a frequently amplified region in chr17q23.2 close to *RPS6KB1* amplifications, and is far apart from the *ERBB2* amplifications in 17q12 (Supplementary Fig. 2). Thus *TLK2* is not co-amplified with *ERBB2.* Supplementary Figure 3a, b shows the copy-number data of *TLK2* and known amplified oncogenes in breast cancer in TCGA^12^ and Metabric^18^ data sets. *TLK2* copy number does not correlate with most known oncogene amplifications, except *RPSKB1* (Pearson *R*=0.796 for the TCGA data set and *R*=0.768 for the Metabric data set). While *Her2* amplifications are enriched in *TLK2*-amplified tumours, their copy numbers do not correlate with each other (*R*=0.187 for TCGA data set and *R*=0.201 for the Metabric data set). This suggests that *TLK2* is frequently co-amplified with *RPS6KB1*, but not with other known amplified genes in breast cancer such as *ERBB2, MYC, CCND1*, and so on. Gene expression data show that *TLK2* expression is primarily upregulated by copy-number increase at this locus (Fig. 1d, *R*=0.81), and correlates with increased tumour stage (Fig. 1e). Among all the breast cancer subtypes, luminal B breast cancers most frequently harbour *TLK2* amplifications (21.3%) (Supplementary Fig. 1b), and also present the highest *TLK2* expression level (Fig. 1e). Here, the Luminal B subtyping is based on the 50-gene PAM50 predictor^19^ using Agilent gene expression data, and is provided by TCGA^12^.

### TLK2 overexpression correlates with worse clinical outcome

To examine the prognostic effect of *TLK2* overexpression (OE), we analysed the available survival data for TCGA breast cancer patients and compared the group of patients with *TLK2*-high tumours versus the rest (see Methods). This revealed a significantly worse overall survival of the *TLK2*-high group (based on log-rank test, *P*=0.040). *TLK2* is mostly amplified in ER+ breast cancers, which are commonly treated with endocrine therapy. To examine if endocrine therapy can eliminate the prognostic effect of *TLK2* OE, we analysed the gene expression data set by Loi *et al*.^20^ for 263 ER+ breast tumours treated with adjuvant tamoxifen monotherapy, which revealed a significantly worse recurrence-free survival of patients with *TLK2*-high tumours (based on log-rank test, *P*=0.001). To further corroborate this finding, we analysed a large gene expression data set for breast cancers with treatment and prognostic information (the Metabric data set)^18^ (Fig. 1f). Among the 220 ER+ breast cancer patients with no adjuvant treatment, those with *TLK2*-high tumours showed significantly worse disease-specific survival than the rest of the patients (based on log-rank test, *P=*0.012). The prognostic effect is preserved in the 389 ER+ breast cancer patients treated with endocrine monotherapy (based on log-rank test, *P=*0.012). Of note, about 93% of the untreated and endocrine-treated ER+ tumours of the Metabric data set are Her2 negative. These data support a poorer outcome of patients with *TLK2*-high tumours irrespective of endocrine therapy.

### Phenotypic changes following ectopic expression of *TLK2*

**Figure 2:**
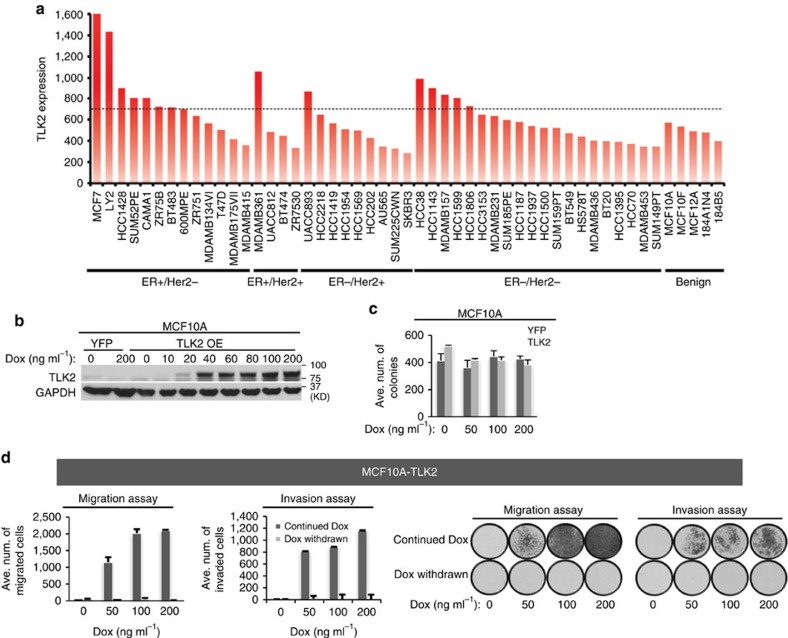
The phenotypic changes after inducible ectopic expression of *TLK2* in the MCF10A benign breast epithelial cells. (**a**) Bar chart showing *TLK2* gene expression in a panel of breast cancer and benign cell lines based on exon expression array data^21^. (**b**) Western blot detecting the TLK2 proteins ectopically expressed in MCF10A cells after induction with different doses of doxycycline (Dox). (**c**) Cell survival following induction of TLK2 expression in MCF10A cells was measured by clonogenic assay. Error bars represent the s.d. of three replicate measurements per condition. (**d**) Transwell migration and matrigel invasion assays. After 2-week induction of TLK2 expression with different doses of Dox, Dox was either continued or withdrawn to test if the increased cell migration and invasion is dependent on TLK2 expression. Error bars represent the s.d. of three replicate measurements per condition.

**Figure 3:**
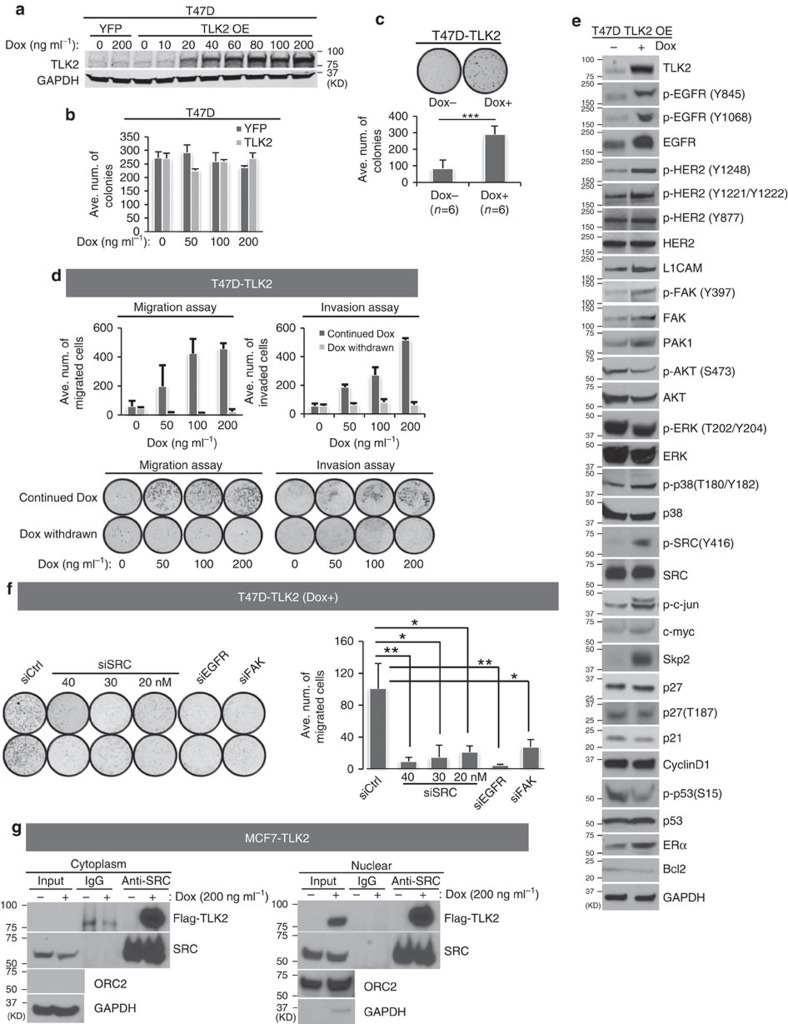
The phenotypic and cell signalling changes after ectopic expression of *TLK2* in the T47D ER+/Her2− luminal breast cancer cells. (**a**) Western blot detecting TLK2 protein ectopically expressed in T47D cells after induction with different doses of Dox. (**b**) Cell survival following induction of TLK2 expression in T47D cells was measured by clonogenic assay. Error bars represent the s.d. of three replicate measurements per condition. (**c**) TLK2 overexpression significantly enhanced anchorage-independent growth of T47D cells. TLK2 was overexpressed in T47D by treating with 100 ng ml^−1^ Dox before the soft-agar assays were performed. Error bars represent the s.d. of three replicate measurements per condition. (**d**) Transwell migration and matrigel invasion assays. Following TLK2 induction for 2 weeks, Dox was either continued or withdrawn to test if the increased cell migration and invasion is dependent on TLK2 overexpression. Error bars represent the s.d. of two replicate measurements per condition. (**e**) Alterations of key signalling molecules in breast cancer were examined by Western blot following TLK2 overexpression in T47D. TLK2 was induced by 200 ng ml^−1^ Dox for 2 weeks. Dox, doxycyclin. (**f**) Transwell migration assays following SRC, EGFR or FAK knockdown in T47D cells overexpressing TLK2. The indicated concentration of siRNAs against SRC or 20 nM of siRNAs against EGFR or FAK were transfected for 3 days following induction of TLK2 expression in T47D cells for two weeks (200 ng ml^−1^ Dox). Western blot validation of SRC, EGFR or FAK silencing in T47D cells overexpressing TLK2 was shown in Supplementary Fig. 5. Error bars represent the s.d. of two replicate measurements per condition. (**g**) Co-immunoprecipitation of TLK2 with SRC in engineered MCF7 cells inducibly expressing Flag-tagged TLK2. Engineered MCF7 cells was treated with 200 ng ml^−1^ Dox for 48 h and nuclear or cytoplasmic proteins were purified following subcellular fractionation. IP was performed using an established monoclonal antibody against SRC after conjugation with agarose beads. Western blot was performed using an anti-Flag antibody to detect the presence of TLK2 in SRC complex. Dox, Doxycyclin.

To assess *TLK2* expression levels in breast cancer cell lines and benign breast epithelial cells, we analysed the Affymetrix exon 1.0 ST expression array data from a previous study^21^. High-*TLK2* expression was observed in multiple breast cancer cell lines (particularly ER+/Her2− lines), but not in benign breast epithelial cells (Fig. 2a). This result was further verified through western blot analysis of a subset of these cell lines (Supplementary Fig. 4a). To determine the transforming activity of TLK2, we selected the MCF10A benign breast epithelial cell line and the *TLK2*-low luminal breast cancer cell line T47D (ER+/Her2−) for the engineering of *TLK2* ectopic OE models. To achieve tuned TLK2 OE, we engineered the coding region of *TLK2* into a doxycycline-inducible lentiviral vector, which was then transduced into these two cell lines (Figs 2b and 3a). Following 2 weeks of TLK2 induction, MCF10A or T47D cells expressing TLK2 did not show significant increase in clonogenic growth compared with the control cells (Figs 2c and 3b). However, soft-agar colony formation assays revealed significantly increased anchorage-independent growth after TLK2 overexpression in T47D cells (Fig. 3c).

To test if TLK2 may enhance cell invasiveness, we gauged the cell motility and invasion capability after TLK2 overexpression using transwell migration and invasion assays. Interestingly, inducible TLK2 overexpression in MCF10A or T47D cells strongly enhanced the cell migration and invasion capabilities in a dose-dependent manner (Figs 2d and 3d). To attribute this to the excess of TLK2 protein, we abolished TLK2 expression by withdrawing the Dox induction in these cell models (Figs 2d and 3d). This eradicated the increased migration and invasion capabilities in both lines, suggesting the dependence of these properties on TLK2 expression itself. These data suggest the role of TLK2 in augmenting cell invasiveness. To examine the cell signalling changes following TLK2 overexpression in the T47D breast cancer cells, we performed western blot analysis of an array of signalling molecules in breast cancer. Interestingly, while the canonical growth factor signalling molecules in breast cancer such as AKT and ERK were not activated with TLK2 overexpression, markedly increased phosphorylation of SRC on Y416 in the activation loop of its kinase domain, as well as modest increased activating phosphorylation of FAK and p38 were observed in the T47D cells overexpressing TLK2 (Fig. 3e). In addition, increased EGFR protein level and phosphorylation at the activating sites (Y845 and Y1068) as well as increased Her2 auto-phosphorylation (Y1248 and Y1221/1222) are observed with TLK2 overexpression (Fig. 3e). Besides these changes, SKP2 protein level was also markedly elevated, and we will discuss later the significance of this alternation. SRC has a key role in breast cancer cell migration, invasion and metastasis^22^,^23^, and activation of the FAK-SRC complex is known to mediate EGF-induced cell motility^24^,^25^. This suggests the possible involvement of the EGFR/SRC/FAK axis in the enhanced invasiveness driven by TLK2.

To access the significance of SRC, FAK and EGFR in TLK2-driven cell motility, we silenced SRC, FAK or EGFR using established siRNAs^26^,^27^,^28^, and assessed transwell cell migration. Interestingly, silencing of either SRC, FAK or EGFR in TLK2-overexpressing T47D cells significantly diminished the cell migration, suggesting that the EGFR/SRC/FAK axis may be involved in the enhanced invasiveness driven by TLK2 (Fig. 3f; Supplementary Fig. 5). To examine if TLK2 interacts with SRC, we performed immunoprecipitation (IP) of cytoplasm or nuclear fractions of the MCF7 cells expressing FLAG-tagged TLK2 using an established monoclonal antibody against SRC^29^,^30^, and detected the presence of TLK2 in SRC complex with anti-FLAG antibody. As a result, we observed the co-precipitation of TLK2 with SRC in both nuclear and cytoplasmic fractions (Fig. 3g). Taken together, these data suggest that EGFR/SRC/FAK axis may be important in TLK2-driven invasiveness in breast cancer cells, and TLK2 may engage this axis via interaction with SRC.

### TLK2 silencing selectively inhibits *TLK2*-high cancer cells

**Figure 4:**
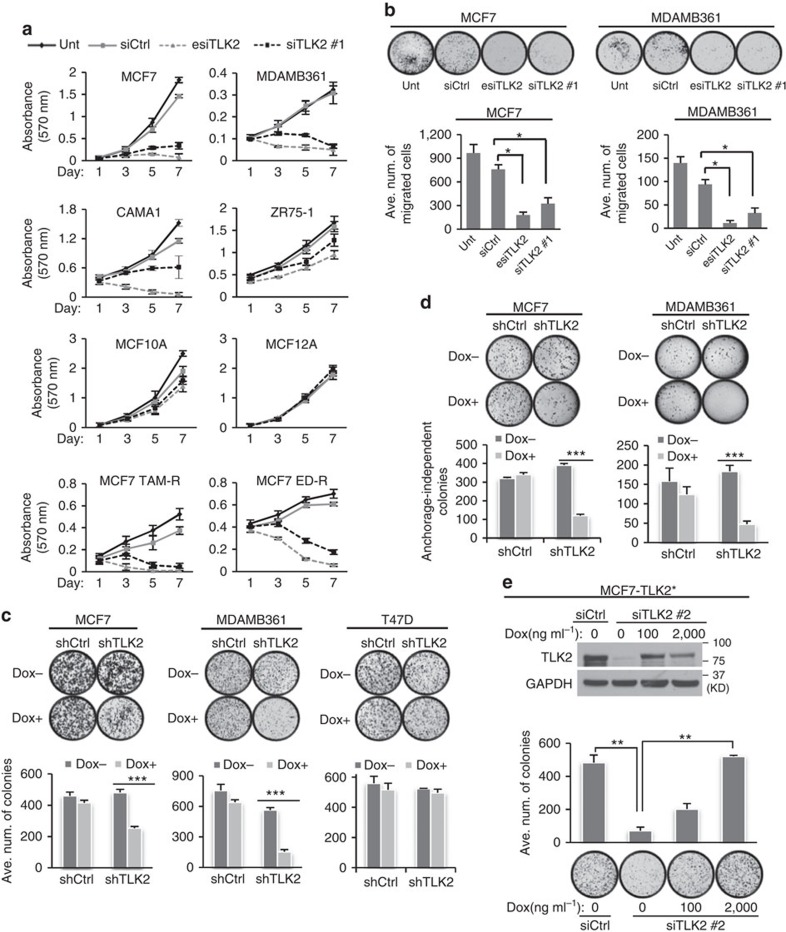
The effect of TLK2 silencing in ER+ breast cancer cells and benign breast epithelial cells. (**a**, **b**) TLK2 knockdown (KD) by esiRNA and siRNA transfection. (**a**) Cell proliferation was assessed by MTT assay in TLK2-high or low breast cancer cell lines or benign breast epithelial cells after TLK2 knockdown. Unt, untreated. Ctrl, control. Error bars represent the s.d. of three replicate measurements per condition. (**b**) Cell migration assessed by Boyden chamber transwell assay following TLK2 KD for 48 h in MCF7 and MDAMB361 cells using TLK2 esiRNA or siRNA. NIH 3T3 cells are used as chemo-attractant for MCF7 and MDAMB361 cells. Error bars represent the s.d. of three replicates measurements per condition. (**c**) Clonogenic assay was performed following Dox-inducible shRNA silencing of TLK2 in breast cancer cells. 0.5 mg ml^−1^ of Dox was used for this assay. Dox, doxycycline. Error bars represent the s.d. of three replicate measurements per condition. (**d**) TLK2 inhibition suppresses anchorage-independent growth of MCF7 and MDAMB361 cells. Dox (0.5 μg ml^−1^) was administered for 2 days to induce TLK2 shRNA and then soft-agar colony formation assay was performed. Error bars represent the s.d. of three replicate measurements per condition. (**e**) The cell growth inhibition after TLK2 KD using a siRNA#2 (with the same targeting sequence as the shTLK2) can be rescued by inducible TLK2 overexpression. Multiple silent mutations at the shTLK2 targeting region are introduced into the *TLK2* ORF without affecting the amino acid sequence, to reduce the inhibition of ectopically expressed TLK2 by siRNA#2. TLK2 expression was induced by treating MCF7 cells with 100 or 2,000 ng ml^−1^ Dox; siTLK2 was then transfected and incubated for 2 weeks for clonogenic assay. Error bars represent the s.d. of two replicate measurements per condition. *P* values were calculated based on *t-*test. *P<0.05; ^**^P<0.01; ^***^P<0.001. Dox, doxycycline.

Next, we went on to examine the effect of TLK2 silencing on breast cancer cell growth. Among the *TLK2*-high breast cancer cell lines, MCF7 and MDAMB361 show the highest *TLK2* expression level (Fig. 2a), and also harbour high levels of *TLK2* amplifications (Fig. 1c). MCF7 and MDAMB361 are both ER+ luminal breast cancer cells, and are negative or positive for Her2, respectively. Thus these two cell lines will be ideal to study the effect of TLK2 inhibition in *TLK2*-amplified luminal breast cancer cells with different Her2 status. To affirm the *TLK2* amplifications in these two cell lines, we performed fluorescence *in situ* hybridization (FISH) using a TLK2-specific probe and a centromere 17 probe (CEN17). As a result, the FISH assay verified *TLK2* amplifications in the MCF7 and MDAMB361 cells with a TLK2:CEN17 ratio of 3.9 and 2.6, respectively (Supplementary Fig. 6). In addition to these *TLK2*-amplified breast cancer cell lines, we also selected a ER+/Her2− luminal breast cancer cell line with moderately high *TLK2* but without *TLK2* amplification (CAMA1), a *TLK2*-low ER+/Her2− luminal breast cancer (ZR75-1), and two benign epithelial cell lines (MCF12A and MCF10A). A MISSION esiRNA (Sigma) against *TLK2* was then introduced into these selected cell lines, and the knockdown efficiency was verified by western blot (Supplementary Fig. 4b, c). The esiRNA is an endoribonuclease prepared siRNA pool that can deliver highly specific and effective gene silencing with lower off-target effects than single or pooled siRNAs^31^. In addition, a TLK2 siRNA#1 targeting a different region from that of the esiRNA was also selected and verified as above (Supplementary Fig. 4b, c). The specificity of these siRNAs against TLK2 but not its paralogue TLK1 was verified by quantitative PCR and western blot (Supplementary Fig. 7a, b). MTT cell proliferation assays revealed that specific silencing of TLK2 by esiRNA or siRNA potently inhibited the growth of *TLK2*-high breast cancer cells (MCF7, MDAMB361 and CAMA1), but not that of the *TLK2*-low luminal ZR75-1 breast cancer cells or MCF12A and MCF10A benign breast epithelial cells (Fig. 4a). Interestingly, the MCF7 derivative strains with acquired resistance to tamoxifen (Tam-R) or oestrogen deprivation (ED-R)^32^ retained high sensitivity to TLK2 inhibition (Fig. 4a), suggesting the potential of TLK2 inhibition in managing these acquired resistant breast tumours. In addition, TLK2 inhibition by both TLK2 esiRNA and siRNA significantly repressed the migration of MCF7 and MDAMB361 cells (Fig. 4b), consistent with our reverse observations in the TLK2 overexpression models.

### Inducible TLK2 inhibition suppresses clonogenic growth

Next, we engineered the MCF7 and MDAMB361 cell lines to inducibly express TLK2 shRNA targeting a region different from those of the TLK2 esiRNA and siRNA (Supplementary Fig. 4b, d), which allowed us to observe the long-term effects of TLK2 inhibition. With induction of TLK2 inhibition, decreased colony-forming ability was observed only in the *TLK2*-high MCF7 and MDAMB361 luminal breast cancer cells, but not in the *TLK2*-low T47D luminal breast cancer cells, as shown by clonogenic assays (Fig. 4c). In addition, we also observed potent inhibition of anchorage-independent growth following TLK2 inhibition in both MCF7 and MDAMB361 cells as shown by soft-agar colony formation assay (Fig. 4d).

To verify the specificity of this TLK2 shRNA, we engineered the MCF7 cells to inducibly express the *TLK2* ORF with multiple silent mutations at the shRNA targeting sites. We then designed a TLK2 siRNA#2 with the same target sequence as the TLK2 shRNA, and introduced it into the MCF7 cells inducibly expressing the mutated *TLK2* ORF, which are subjected to clonogenic assays with or without Dox induction. Ectopic expression of TLK2 resulted in a significant rescue of the knockdown effect by TLK2 siRNA#2 in a dose-dependent manner, which validates the specificity of the TLK2 shRNA (Fig. 4e).

### The effect of TLK2 inhibition in a xenograft tumour model

**Figure 5:**
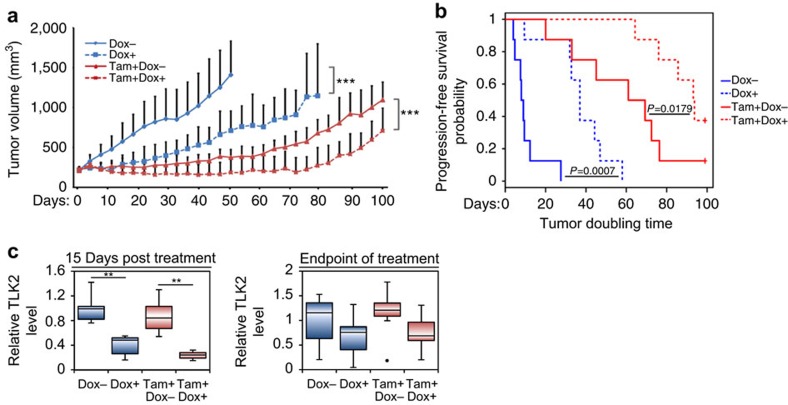
The therapeutic effect of TLK2 inhibition in a MCF7 preclinical xenograft tumour model. (**a**) The effect of TLK2 inhibition in the MCF7 xenograft tumours inducibly expressing a TLK2 shRNA, in the presence or absence of concomitant tamoxifen treatment. The average tumour growth in each treatment group (8 mice per group). Error bars represent the s.d. of tumour volumes of 8 mice measurements per condition. *P* values were calculated based on ANOVA to compare the tumour volumes. (**b**) Kaplan–Meier survival plot comparing the progression-free survival of different treatment groups (based on tumour-doubling time). Generalized Wilcoxon test was used to calculate the *P* values for comparing progression-free survival between different treatment groups. (**c**) Quantitative western blot analysis of TLK2 protein expression in the tumours harvested after 15 days of treatment (5 mice/group), or at the end point (8 mice per group). Error bars represent the s.d. of relative TLK2 levels of 5 or 8 mice measurements per condition. *P<0.05; ^**^P<0.01; ^***^P<0.001. Dox, doxycycline. Corresponding western blot images are shown in Supplementary Fig. 8.

To examine the therapeutic effect of TLK2 inhibition in a preclinical mouse model, we transplanted the MCF7 cells inducibly expressing the TLK2 shRNA into female athymic nude mice, and assessed the potential therapeutic effect of TLK2 inhibition in the *in vivo* context (Fig. 5a). As most ER+ breast tumours are treated with endocrine therapy, we also examined the combination effect of TLK2 inhibition together with tamoxifen, the most commonly used endocrine agent. Upon tumour establishment, mice were randomized and treated with vehicle or tamoxifen (Tam), and further subdivided into ±doxycycline treatments. To verify the effectiveness of TLK2 inhibition, a subset of tumours were harvested after 2 weeks of treatment, and analysed by western blot. To observe relative long-term therapeutic effects, the rest of the mice were monitored for up to 100 days depending on the duration of tumour control. Our result showed that TLK2 inhibition alone or in combination with tamoxifen substantially inhibited the growth of MCF7 xenograft tumours. Kaplan–Meier survival analysis revealed a significant increase in progression-free survival in TLK2 inhibition alone or concomitant TLK2 inhibition and tamoxifen treatment groups compared with the control groups (Fig. 5b). While the tumours in the combined treatment group still re-grew after 70 days of treatment, this could be attributable to the loss of TLK2 inhibition as suggested by the western blot analysis of tumours harvested at the end of the treatments (Fig. 5c; Supplementary Fig. 8). Such loss of target inhibition in the inducible knockdown tumour model after long-term induction of shRNA expression may be due to the selective pressure imposed by the target inhibition, which has been observed by others as well^33^. Together, these data provided a proof of concept for the therapeutic value of TLK2 inhibition in *TLK2*-amplified breast cancers.

### The signalling changes following TLK2 inhibition

**Figure 6:**
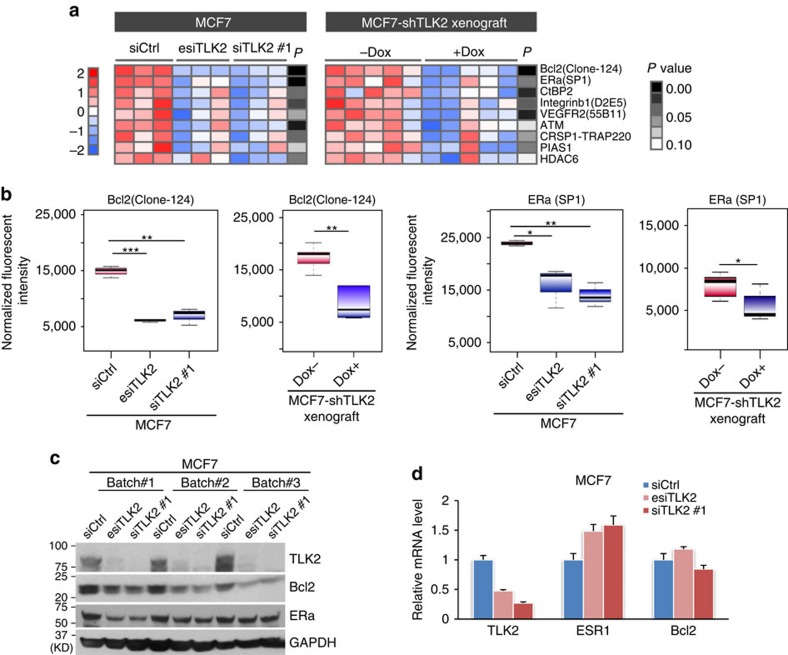
RPPA profiling results after TLK2 knockdown in MCF7 cells or MCF7 xenograft tumours inducibly expressing shTLK2. (**a**) The heat-map of consistently altered signalling proteins revealed by RPPA analyses of MCF7 cells ±TLK2 KD and MCF7 xenograft tumours harvested 2 weeks after induction of TLK2 inhibition. Proteins that showed a consistent trend of changes (*P*<0.1) in both *in vitro* and *in vivo* models are shown in the heat-map, and are sorted by the mean *P* values of different comparisons. For RPPA profiling, each knockdown experiment was repeated three times biologically. *P* values were calculated based on *t-*test and are shown in grey scale. (**b**) Boxplots of normalized fluorescent intensities of Bcl2 or ERα after TLK2 knockdown in MCF7 cells (3 repeats for each group) or MCF7 xenograft tumours (5 tumours in each group). The whiskers indicate the max and min values and horizontal lines represent the 1st, 2nd and 3rd quartiles. (**c**) Western blot validation of Bcl2 and ERα protein changes after TLK2 knockdown in MCF7 cells. (**d**) Q-PCR results quantifying relative mRNA level of TLK2, ERα, or Bcl2 after TLK2 silencing by transfecting MCF7 cells with 10 nM of siCtrl, esiTLK2, or siTLK2 #1. Error bars represent the s.d. of three replicate measurements per condition.

To systematically profile the cell signalling changes after TLK2 inhibition, we performed reverse phase protein array (RPPA) analysis of the MCF7 cells treated with TLK2 siRNAs as well as the xenograft tumours harvested after two weeks of TLK2 inhibition, using 200 validated antibodies against an array of key signalling molecules in cancer. We then isolated the consistently altered signalling molecules across different *in vitro* and *in vivo* TLK2 knockdown models based on student's *t*-test at 90% confidence (Fig. 6a; Supplementary Fig. 9). This revealed several consistently altered signalling molecules after TLK2 inhibition, among which BCL2 and ERα are the most significantly down-regulated proteins (based on *t*-test, the average *P* value for BCL2 is 0.0005, and average *P* value for ERα is 0.0252) (Fig. 6a, b). The downregulation of BCL2 and ERα after TLK2 knockdown were further verified by western blots (Fig. 6c). To test if these effects may be due to the off-target effects of TLK2 siRNAs, we examined the expression of BCL2 and ERα transcripts following TLK2 knockdown by quantitative PCR, which revealed no significant change or even upregulation of the mRNA levels of BCL2 and ERα (Fig. 6d). This suggests that the modulations of BCL2 and ERα by TLK2 knockdown are mainly at the protein level. To examine if TLK2 overexpression upregulates BCL2 and ERα protein level in breast tumour tissues, we compared the BCL2 and ERα protein level in ER-positive breast cancers with or without TLK2 overexpression using the RPPA data available from TCGA (Supplementary Fig. 10). As a result, we found that ERα protein is significantly elevated in *TLK2*-high breast tumours (based on *t*-test, *P*=0.041), in spite of a slight increase of ESR1 transcript (based on *t*-test, *P*=0.158). While both BCL2 transcript and protein are slightly higher in *TLK2*-high tumours, such differences are not statistically significant. This suggests that TLK2 may play a role in modulating ERα protein level in breast tumours, whereas the BCL2 response may be an effect specific to TLK2 inhibition.

### TLK2 silencing impedes G1/S transition and induces apoptosis

**Figure 7:**
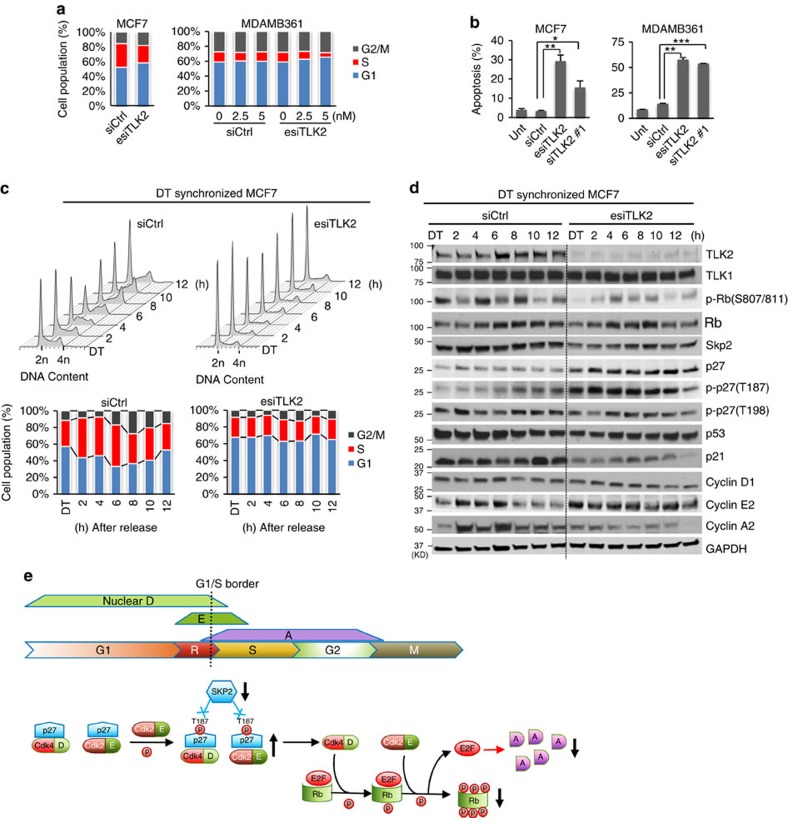
TLK2 inhibition results in impaired G1/S progression and induction of apoptosis in TLK2-amplified luminal breast cancer cells. (**a**) Flow cytometry results showing cell cycle changes after TLK2 knockdown in asynchronized MCF7 and MDAMB361 cells. 10 nM siRNAs (for MCF7 cells) or indicated concentration of siRNAs (for MDAMB361 cells) were transfected for 72 h. Ctrl, Control. (**b**) Cell apoptosis assessed by Annexin V assay in asynchronized MCF7 and MDAMB361 cells following TLK2 knockdown via 20 nM esiTLK2 or siTLK2#1 transfection for 72 h. Error bars represent the s.d. of two replicate measurements per condition. *P* values are calculated based on *t-*test. *P<0.05; ^**^P<0.01; ^***^P<0.001. (**c**) Cell cycle profile of synchronized MCF7 cells (by double thymidine block) after TLK2 inhibition. After TLK2 knockdown for 24 h, MCF7 cells were synchronized at the G1/S border using 2.5 mM double thymidine (DT), and then released. Cells were collected every 2 h after cell cycle release for up to 12 h, and analysed for DNA content using flow cytometry. (**d**) Western blot was done to examine the changes of key signalling molecules involved in G1/S cell cycle regulation using the cell lysates obtained from the same experiment as in 7c. ‘DT' indicates synchronized MCF7 cells by DT block. (**e**) A schematic of normal G1/S cell cycle signalling and alternations following TLK2 inhibition (black arrows). In normal cell cycle, the cyclin E level starts to increase in late G1 phase, and then collapses as the cells enter S phase^53^, followed by increased cyclin A expression^54^,^55^. Rb regulates G1/S transition by repressing the E2F transcription factors that control the expression of cyclin A. Once Rb is phosphorylated (that is, at S807/S811), it releases E2Fs, which will allow cells to enter S phase^56^,^57^. p27 inhibits the two G1 cyclin/cdk complexes, cyclin D/Cdk4 and cyclin E/Cdk2 (refs 36, 40), both of which are the key upstream kinases of Rb (ref. 37). During normal G1/S progression, the p27 proteins complexed with G1 cyclin/cdks were phosphorylated by the p27-free cyclin E/Cdk2 complexes at T187, which were then targeted for SKP2-mediated proteasome degradation^58^. D, Cyclin D. E, Cyclin E. A, Cyclin A. R, restriction point.

To examine the impact of TLK2 inhibition on cell cycle progression, we performed flow cytometry of DNA content after TLK2 knockdown in asynchronized MCF7 and MDAMB361 cells. As shown in Fig. 7a, TLK2 knockdown led to substantial increase of G1 phase cells and decrease of S-phase cells, suggesting delayed cell cycle progression through the G1/S border. TLK2 inhibition also potently induced apoptosis in MCF7 and MDAMB361 cells, as shown by Annexin V assay (Fig. 7b). To observe the dynamic cell cycle progression after TLK2 knockdown, we performed a series of flow cytometry analyses following TLK2 knockdown by esiRNA in the MCF7 cells synchronized with a double thymidine (DT) block (Fig. 7c). Consistently, we observed delayed cell cycle progression through the G1/S border. In addition, western blot analysis revealed sustained high cyclin E level and low cyclin A level in response to TLK2 inhibition after cell cycle release from the DT block (Fig. 7d), suggesting that these cells were hindered from progressing into S-phase (Fig. 7e).

In addition, we also observed a markedly increased p27 protein level, and a decreased level of SKP2, the key E3-ligase of p27 (Fig. 7d)^34^. p27 inhibits the cyclin D/Cdk4 and cyclin E/Cdk2 complexes, and blocks cell cycle progression through the G1/S border^35^. Thus the impeded G1/S transition may be attributable to increased p27 level. Interestingly, enhanced phosphorylation of p27 at T187 was also observed with TLK2 silencing. The phosphorylation of T187 is known to target p27 to the SCF^Skp2^ ubiquitin ligase complex and proteasome-mediated degradation^36^. This suggests that the increased p27 protein level may be attributable to the decrease in SKP2, the key E3-ligase of p27, instead of impaired T187 phosphorylation. Furthermore, we also observed a decrease in phosphorylated Rb (S807/S811) after TLK2 inhibition. Since p27 inhibits cyclin D/Cdk4 and cyclin E/Cdk2, the key upstream kinases of Rb (ref. 37), the increased p27 after TLK2 knockdown may prevent Rb phosphorylation and subsequent E2F release^38^.

To verify the above results, we synchronized the MCF7 cells at M phase via a nocodazole block, and the cell cycle was released in the condition of TLK2 inhibition by the siRNA#1 that targets a different region from TLK2 esiRNA (Supplementary Fig. 4b). Cells were then subjected to flow cytometry analyses (Supplementary Fig. 11). The nocodazole mediated mitotic block allowed observing the cell cycle progression from M to G1, then to S-phase. Consistent with the DT block results, TLK2 inhibition delayed cell cycle progression through the G1/S border after MCF7 cells were released from mitotic arrest.

### Differential cellular response to TLK2 or TLK1 inhibition

**Figure 8:**
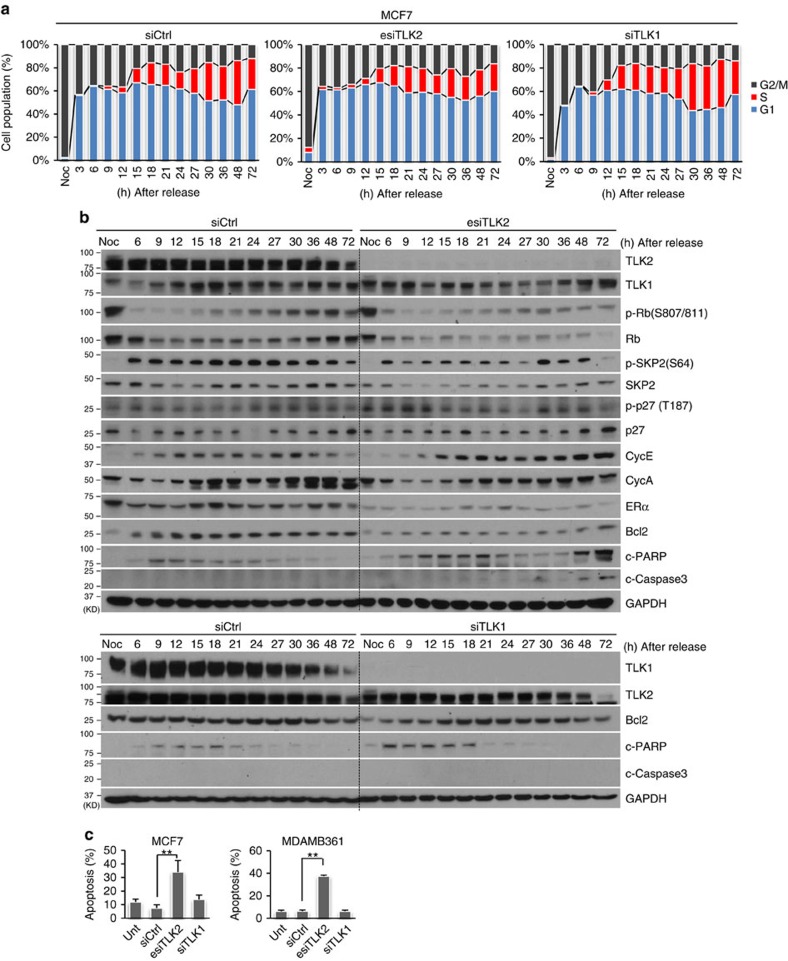
*TLK2*-amplified luminal breast cancer cells respond differentially to TLK2 or TLK1 inhibition. (**a**) Cell cycle profile of MCF7 cells synchronized by nocodazole block after TLK2 or TLK1 knockdown. After TLK2 or TLK1 silencing by transfecting 10 nM of esiTLK2 or siTLK1 for 24 h, MCF7 cells were synchronized at mitosis using 200 nM nocodazole for 15 h, and then released. Cells were collected at the indicated time after cell cycle release. To precisely determine S-phase cell population, 10 μM of BrdU was added for 1.5 h before cell collection. The cell cycle distributions were determined based on DNA content and BrdU incorporation (Supplementary Fig. 12). (**b**) Western blot was done to examine the changes of key signalling molecules involved in G1/S cell cycle regulation and apoptosis using the cell lysates obtained from same experiment as in Fig. 8a. ‘Noc' indicates the MCF7 cells synchronized at mitosis by nocodazole block (before cell cycle release). (**c**) Cell apoptosis assessed by Annexin V assay in asynchronized MCF7 and MDAMB361 cells following 20 nM of esiTLK2, siTLK1, or siCtrl treatment for 72 h. Error bars represent the s.d. of two replicate measurements per condition. *P* values are calculated based on *t-*test. ^**^P<0.01.

To observe the different roles of TLK2 and TLK1 in cell cycle regulation, we performed comparative cell cycle analyses of nocodazole-synchronized MCF7 cells in the condition of either TLK2 or TLK1 inhibition. TLK2 inhibition was accomplished using TLK2 esiRNA, whereas TLK1 was silenced using a specific siRNA previously documented^39^. To precisely determine the S-phase cell population, we incubated the cells with 5-bromo-2'-deoxyuridine (BrdU), the S-phase DNA synthesis marker, before cell collection. Cells were then subjected to flow cytometry analysis, and cell cycle distribution was determined based on both DNA content and BrdU incorporation (Supplementary Fig. 12). In addition, cell signalling changes were monitored for up to 72 h to observe if there is enhanced apoptosis following impaired G1/S progression. Interestingly, while TLK2 inhibition led to delayed G1/S progression, TLK1 repression resulted in delayed S-phase progression, consistent with its known function in promoting chromatin assembly during S-phase (Fig. 8a)^7^. This suggests the distinct roles of TLK1 and TLK2 in cell cycle regulation.

Consistent with the previous data, western blots showed downregulation of SKP2, upregulation of p27, as well as increased cyclin E and decreased cyclin A levels after TLK2 inhibition (Fig. 8b). Further, we also observed repression of BCL2 and ERα after TLK2 inhibition. BCL2 is an anti-apoptotic factor overexpressed in MCF7 cells^40^. Consistent with BCL2 repression, increased cleavage of caspase 3 and of PARP was observed in the TLK2-repressed MCF7 cells, suggesting induction of apoptosis. In contrast, inhibition of its paralog TLK1 did not significantly affect the BCL2 protein level, and there is no significant induction of cleaved caspase 3 or PARP (Fig. 8b, lower panel). To further verify this observation, we performed Annexin V assays after TLK2 or TLK1 silencing in MCF7 and MDAMB361 cells. While TLK2 inhibition significantly induced apoptosis, there was no significant increase in apoptosis after TLK1 silencing (Fig. 8c).

Taken together, these data suggest that, while TLK1 and TLK2 are close paralogs, these two kinases may play different roles in cell cycle regulation. Silencing of TLK1 or TLK2 appears to result in distinct cell cycle alterations and different effects on apoptosis in luminal breast cancer cells overexpressing TLK2.

### A kinase profiling data set reveals potential TLK2 inhibitors

**Figure 9:**
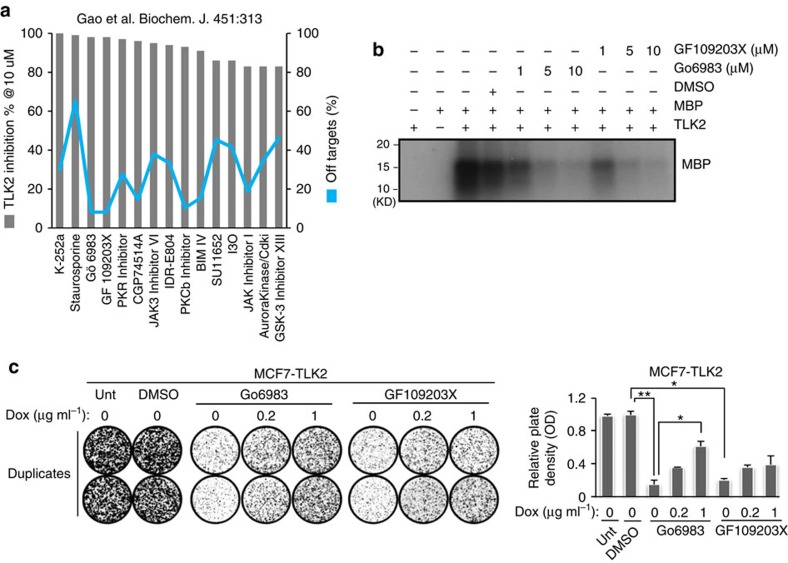
Characterizing potential TLK2 inhibitors identified from a public kinase profiling data set. (**a**) Nominating potential TLK2 inhibitors based on analysis of a public kinase profiling data set^41^. Two potential TLK2 inhibitors were selected based on relatively strong TLK2 inhibition (shown in bar chart) and low number of off-targets (shown in line chart). (**b**) The activity of the potential TLK2 kinase inhibitors Go6983 and GF109203X against TLK2 kinase were measured by *in vitro* kinase assay using recombinant active TLK2 protein. Myelin Basic Protein (MBP) was used as a substrate. (**c**) Clonogenic assays following Go6983 or GF109203X treatment of the MCF7 cells inducibly expressing TLK2. Go6983 (4 μM) or GF109203X and 0.2 or 1 μg ml^−1^ of Dox were administered. Error bars represent the s.d. of two replicate measurements per condition. *P* values are calculated based on *t-*test. *P<0.05; ^**^P<0.01.

To identify potential TLK2 inhibitors, we investigated a publicly available kinase profiling data set that profiled the activity of 158 structurally diverse kinase inhibitors against 234 recombinant protein kinases^41^. We ranked these kinase inhibitors based on their activities against the TLK2 kinase, and evaluated their potential off-target effects based on the number of kinases against which the inhibitors presented stronger activities than TLK2 (Fig. 9a). Interestingly, two PKC inhibitors Go6983 and GF109203X were found to possess relatively strong and selective activities against TLK2. Both compounds are ATP-competitive inhibitors that bind to the ATP pocket of the PKC kinase catalytic domain. Kinase profiling data suggest that both Go6983 and GF109203X inhibit ∼98% of TLK2 kinase activity at 10 μM. We therefore performed *in vitro* kinase assays with myelin basic protein as a substrate, using recombinant active TLK2 proteins (SignalChem) treated with different doses of Go6983 or GF109203X (Fig. 9b). Both compounds resulted in potent inhibition of TLK2 activity at 5 or 10 μM. To assess their therapeutic effects via TLK2, we dosed MCF7 cells inducibly expressing TLK2 with 4 μM Go6983 or GF109203X, and measured cell viability via clonogenic assays. Both compounds strongly inhibited cell viability, whereas induction of TLK2 overexpression can partially rescue the effect in a dose-dependent manner (Fig. 9c). This suggests that the therapeutic effects of these PKC inhibitors are at least partially via their actions against TLK2. Between these compounds, Go6983 showed a better inhibitory effect on cell viability and a stronger rescue effect from TLK2 overexpression. While Go6983 and GF109203X may not be applicable *in vivo* due to their off-target effects and the requirement of high doses to sufficiently inhibit TLK2 activity, these compounds could possibly serve as backbones for the future development of more potent and specific TLK2 inhibitors.

## Discussion

In this study, ConSig-Amp analysis nominated *TLK2* as a candidate kinase target upregulated by genomic amplifications in more aggressive form of luminal breast cancers. *TLK2* amplification is independent of most known amplified oncogenes in breast cancer (that is, *HER2, CCND1* and *MYC*), except *RPSKB1* (Supplementary Fig. 3). While *TLK2* is often co-amplified with *RPSKB1* due to their vicinity (Supplementary Fig. 2), it is not uncommon that multiple closely located oncogenes are targeted by the same genomic amplifications in breast cancers, such as the co-amplifications of *ERBB2* and *GRB7* (ref. 42), *FGFR1* and *WHSC1L1* (ref. 43), or *PAK1* and *GAB2* (ref. 44). In fact, genomic amplifications in cancer usually affect multiple genes in the amplified regions. Besides luminal breast cancer cells, *TLK2* is also overexpressed in a few ER-negative breast cancer cell lines (Fig. 2a). However, these cell lines typically do not harbour high *TLK2* amplifications, and TCGA copy-number data suggest that *TLK2* amplifications are much more frequent in ER-positive than negative breast cancers, 10.5% versus 2.9% (Supplementary Fig. 1b). Consistently the latest phosphoproteomic study of TCGA breast tumours by The Clinical Proteomic Tumour Analysis Consortium (CPTAC) independently identified TLK2 as an amplicon-associated highly phosphorylated kinases in luminal breast cancer^11^, which further support the significance of *TLK2* amplification and its preferential association with luminal tumours. Our study is the first comprehensive analysis of TLK2 function in aggressive luminal breast cancers, which will timely complement the CPTAC paper. Our data showed that ectopic overexpression of TLK2 in the T47D luminal breast cancer cells markedly increased cell migration and invasion, whereas withdrawal of TLK2 expression eliminated this effect, suggesting the direct role of TLK2 in enhanced invasiveness. Furthermore, we found that TLK2 may involve the EGFR/SRC/FAK axis to enhance breast cancer cell invasiveness (Fig. 3e–g). Future studies will be needed to understand the precise mechanisms of TLK2-driven cell invasiveness and how exactly TLK2 interacts with the EGFR/SRC/FAK axis.

More important, breast cancer cells that harbour *TLK2* amplifications appear to have been addicted to TLK2 overexpression, so that TLK2 knockdown causes potent growth inhibition and induction of apoptosis. In addition, we observed a selective effect of TLK2 inhibition on *TLK2*-high breast cancer cells versus *TLK2*-low breast cancer cells or benign breast epithelial cells. Of note, these effects appear to be sustained in the breast cancer cells that have already developed resistance to endocrine therapy. Our mechanistic studies suggest that TLK2 inhibition downregulates SKP2, upregulates p27, and impedes cell cycle progression through the G1/S border. In addition, we found that TLK2 inhibition consistently suppresses ERα and BCL2 protein level *in vitro* and *in vivo*, which may contribute to the substantially decreased cell proliferation and enhanced apoptosis after TLK2 inhibition. In contrast, inhibition of the TLK2 paralog, TLK1, resulted in a delay in S-phase progression, and did not significantly induce apoptosis (Fig. 8). This is the first observation on a role of TLK2 distinct from that of TLK1 in regulation of cell cycle progression—the latter (TLK1) has been known to promote chromatin assembly during S-phase.

It is interesting to note that ectopic expression of TLK2 in T47D cells did not increase cell proliferation (Fig. 3b). While TLK2 overexpression upregulates SKP2, the key E3-ubiquitin ligase of p27 (ref. 45), we found that p27 protein level is not significantly down-regulated. This suggests that additional prerequisites may be needed to fulfill TLK2 function in modulating p27 and G1/S cell cycle progression. One possibility is that *RPS6KB1* is frequently co-amplified with *TLK2* (Supplementary Fig. 2), which may provide additional genetic background for the action of endogenously overexpressed TLK2 in breast cancer cells. Alternatively, the microenvironment of the primary tumours may provide additional signalling required for the action of endogenous TLK2. This reflects the limitations of ectopic expression cell line models in studying the action of amplified oncogenes, as such models may not possess the genetic background or microenvironment required for the full actions of endogenously amplified oncogenes, and therefore may not faithfully reproduce the mechanistic and phenotypic aspects of the oncogenes.

Furthermore, our *in vivo* data suggest that TLK2 inhibition may possess viable therapeutic value in *TLK2*-amplified luminal breast tumours. As shown by the study of a preclinical xenograft tumour model, TLK2 inhibition significantly improved progression-free survival. Nevertheless, we admit the limitations of our *in vitro* and *in vivo* cell line models on predicting therapeutic values, due to excessive clonal evolutions of *in vitro* cultured cells and lack of stromal interactions. Future studies will be required to further evaluate the therapeutic effect of TLK2 inhibition in patient-derived xenograft tumour models, and ultimately in clinical trials of breast cancer. Of note, *TLK2* appears to be more frequently amplified than other known cell cycle kinases or checkpoint kinases^46^ in breast cancers as shown by TCGA copy-number data (Supplementary Table 3). The selective effect of TLK2 inhibition against *TLK2*-high breast cancer cells as compared with benign breast epithelial cells suggests a possible more selective cellular effect of TLK2 inhibitors as compared with the inhibitors of other cell cycle kinases that do not show cancer cell specificity. Moreover, we have identified two potential TLK2 inhibitors and tested their therapeutic activities against TLK2 *in vitro*. These compounds could serve as backbones for future drug development. Taken together, these facts position TLK2 as an attractive cell cycle kinase target for more aggressive luminal breast cancers that harbour *TLK2* amplifications.

## Methods

### Integrative ConSig-amp analysis

To discover new therapeutic targets in ER+ breast cancer, we analysed the copy number (Affymetrix SNP 6.0) and RNAseq (UNC RNAseqV2) data sets available for breast tumours from The Cancer Genome Atlas Project (TCGA)^12^. Normalized ‘level 3' data (segmented by the CBS algorithm) (14) were directly applied in the analysis. First, the copy-number segments were matched with human genes based on physical coordinates to obtain gene-level copy-number data. The frequency of genomic amplification of each human gene in breast cancer was assessed; breast tumours with relative copy number at the respective gene locus more than 0.7 were considered as amplification positive. Genes that are amplified in >5% of ER+ tumours were nominated, and their expressions based on RNAseq data were correlated with copy-number data by Spearman's correlation statistics. The druggability of these genes was predicted based on a drug-target database compiled from multiple sources^13^,^14^,^15^. Then all candidates were ranked by the ConSig-amp score calculated by multiplying the Spearman's correlation coefficient by the concept signature (ConSig) score that we have developed that prioritizes functionally important genes underlying cancer by accessing their associations with cancer-related molecular concepts^2^. The ConSig scores are calculated using a cancer gene list (*n*=385) compiled from the Cancer Gene Census (http://www.sanger.ac.uk/genetics/CGP/Census) and the Mitelman database (http://cgap.nci.nih.gov/Chromosomes/Mitelman), and a compiled molecular concept database including the C1, C2, C3 and C5 gene sets from MSigDb (http://www.broadinstitute.org/gsea/msigdb), and gene interactions from NCBI (ftp://ftp.ncbi.nlm.nih.gov/gene/GeneRIF) and Visant (http://visant.bu.edu/) databases. The detailed protocol to calculate the ConSig Score and the precomputed scores used in this study (for all human genes) are available in the website http://consig.cagenome.org (release 2). The top 50 druggable candidate oncogenes amplified in ER+ breast cancers are provided in Supplementary Table 1 (ranked based on ConSig-amp score). The ConSig-amp scores range from 0 to 2.5. The ConSig-amp scores for *ERBB2, PTK2, RPSKB1* and *TLK2* are 2.49, 2.45, 1.94 and 1.55 respectively.

### Gene expression data and survival analysis

To examine the prognostic value of TLK2 overexpression in ER-positive breast cancer, we analysed the overall survival data available for TCGA patients and correlated with the *TLK2* gene expression data obtained from the level 3 RNAseq data. In addition, we also analysed the survival gene expression data sets by Loi *et al*. (GSE6532, Affymetrix U133 plus v2.0)^20^, and Molecular Taxonomy of Breast Cancer International Consortium (Metabric data set, Illumina HT-12 v3)^18^. Normalized gene expression data matrixes were used for survival analysis. To select optimal TLK2 probes for survival analysis, we aligned the TLK2 probes in the Illumina HT-12 v3 and Affymetrix U133 arrays with human reference genes. In the Illumina HT-12 v3 array, ILMN_1663486 is the only probe that specifically aligns to *TLK2* but not to *TLK2* homologues. In the Affymetrix U133 arrays, 212986_s_at and 212997_s_at are the only TLK2-specific probes; thus the mean of these two probes was used for subsequent survival analysis. All these TLK2-specific probes map to the last exon of *TLK2*. ILMN_1663486 is within the Affymetrix 212997_s_at probe region. Patients were divided into two groups (TLK2 high and the rest) based on the cutoff of median+1 × MAD (median absolute deviation). MAD is calculated using the R with default constant. Kaplan–Meier analyses were carried out using the R survival package. Follow-up time was constrained to a maximum of 10 years. *P* values were calculated based on the log-rank test (*P* values were not adjusted for multiple comparisons). PAM50-based clinical subtypes of breast cancer for TCGA samples were derived from the UCSC Cancer Genome Browser (https://genome-cancer.ucsc.edu/)^47^,^48^. For the Affymetrix Human Exon 1.0 ST data for breast cancer cell lines^21^, exon expression signals were extracted using the RMA-sketch of Affymetrix power tools. *TLK2* gene expression signals were summarized by taking the mean of the expression values of the probes mapping to the last exon of *TLK2*.

### Cell culture

T47D, MDAMB361, CAMA1, ZR75-1, MCF10A and MCF12A cells were obtained from American Type Culture Collection (ATCC) included in the NCI-ATTC ICBP 45 cell line kit. 293FT cells used for lentivirus packaging were purchased from Invitrogen. MCF7 cells, a tamoxifen-resistant MCF7 clone (MCF7 TAM-R), and an oestrogen deprivation-resistant MCF7 clone (MCF ED-R) were obtained from Dr Rachel Schiff's lab^32^. MCF7 and T47D cells were cultured in RPMI 1640 (Cellgro) with 10% fetal bovine serum (Thermo Fisher Scientific). MDAMB361 and 293FT cells were cultured in DMEM (Thermo Fisher Scientific) with 10% fetal bovine serum. MCF10A and MCF12A were cultured as described^49^. MCF7 Tam-R and ED-R cells were maintained in phenol red-free RPMI 1640 (Corning) containing 10% charcoal-dextran treated fetal bovine serum (CD-FBS, Thermo Fisher Scientific), and to sustain the tamoxifen resistance, 10^−7^ M tamoxifen was added to MCF7 TAM-R cells.

### siRNA or esiRNA transfection

The TLK2-specific esiRNA (#EHU113941), customized TLK2 siRNA#2 (5′-CCCAGAAUAGUUAAGCUGU-3′), TLK1 siRNA (#SIHK2292), and control siRNAs (#SIC001) were purchased from Sigma-Aldrich. In addition, customized TLK2 siRNA#1 (5′-GAUAGAAAGACAACGGAAA-3′), SMARTpool EGFR siRNA (E-003114-00-0005, #1 5′-GUCUUAUCUAACUAUGAUG-3′, #2 5′-UCACUCUCCAUAAAUGCUA-3′, #3 5′-GUAACAAGCUCACGCAGUU-3′, #4 5′-GGAUAUUCUGAAAACCGUA-3′), FAK ( 5′-AACCACCUGGGCCAGUAUUAUUU-3′), SRC siRNA (J-003175-16-0005, 5′-GGGAGAACCUCUAGGCACA-3′) and control siRNA (D-001810-10-20) were purchased from Dharmacon. For transfection, 10–20 nM esiRNA or siRNA was applied using Lipofectamine RNAi MAX (Invitrogen) according to manufacturer's instructions.

### MTT cell proliferation assay

Cells (1,000–3,000) were seeded in 96-well plates 24 h before the siRNA or esiRNA transfection. Cell proliferation was analysed for 7 days by MTT assay using the Cell Proliferation kit I (Roche) following manufacturer's protocols.

### Western blot

Cells were extracted in RIPA lysis buffer (Sigma-Aldrich), supplemented with complete protease inhibitor cocktail tablet (Roche). Protein samples were separated in SDS-PAGE gel and then transferred onto a 0.2 μm nitrocellulose membrane. Primary antibodies were used with 1:500-1:2,000 dilution. The following antibodies were used for western blot: rabbit anti-TLK2 (Bethyl Laboratories, A301-257A, 1:1,000), rabbit anti-TLK1 (Bethyl Laboratories, A301-252A, 1:1,000), mouse anti-GAPDH (Santa Cruz, sc-32233, 1:2,000), rabbit anti-phospho p27 (T187) (Abcam, ab75908, 1:500), mouse anti-L1CAM (Abcam, ab3200, 1:1,000), rabbit anti-phospho p27 (T198) (Abcam, ab64949, 1:500), rabbit anti-p27 (Santa Cruz, sc-528, 1:500), rabbit anti-Cyclin A2 (Santa Cruz, H-432, 1:2,000), mouse anti-Bcl2 (Dako, M0887, 1:1,000). Rabbit anti-Cyclin D1 (#2978, 1:1,000), rabbit anti-Cyclin E2 (#4132, 1:1,000), rabbit anti-Skp2 (#2652, 1:1,000), rabbit anti-phopho-p53 (S15) (#9284, 1:1,000), rabbit anti-p53 (#9282, 1:1,000), rabbit anti-p21 (#2947, 1:1,000), rabbit anti-ERα (#8644, 1:1,000), rabbit anti-EGFR (#4267, 1:1,000), rabbit anti-phospho EGFR (Y845) (#6963, 1:1,000), rabbit anti-phospho EGFR (Y1068) (#3777, 1:1,000), rabbit anti-phopho FAK (Y397) (#8556, 1:500), rabbit-anti FAK (#13009, 1:1,000), rabbit anti-PAK1 (#2602, 1:1,000), rabbit anti-HER2 (#4290, 1:1,000), rabbit anti-phospho HER2 (Y1248) (#2247, 1:1,000), rabbit anti-phospho HER2 (Y1221/1222) (#2243, 1:1,000), rabbit anti-phospho HER2 (Y877) (#2241, 1:1,000), rabbit anti-phospho Rb (S807/811) (#8516, 1:1,000), mouse anti-Rb (#9309, 1:1,000), rabbit anti-SRC (#2123, 1:1,000), rabbit anti-phospho SRC (Y416) (#6943, 1:1,000), rabbit anti-phospho AKT (S473) (#4060, 1:1,000), rabbit anti-AKT (#4691, 1:1,000), rabbit anti-phospho ERK1/2 (T202/Y204) (#4370, 1:1,000), rabbit anti-ERK1/2 (#4695, 1:1,000), rabbit anti-phospho p38 (T180/Y182) (#4511, 1:1,000), rabbit anti-p38 (#8690, 1:1,000), rabbit anti-phospho c-Jun (S63) (#9261, 1:1,000), rabbit anti-c-myc (#13987, 1:1,000), rabbit anti-c-Caspase 3 (#9661, 1:500), and rabbit anti-c-PARP (#5625, 1:1,000) were purchased from Cell Signalling. Uncropped western blots were shown in Supplementary Fig. 13.

### Reverse-transcription PCR and quantitative PCR

Total RNA was isolated with the RNeasy Mini Kit (Qiagen) according to the manufacturer's protocol. Complementary DNA was synthesized from 1 μg total RNA, using a Transcriptor First Strand cDNA Synthesis Kit (Roche) in the presence of both oligo (dT) and random primers. The sequences of all PCR primers are listed in Supplementary Table 2. The relative expression level of each target gene was determined using the comparative threshold cycle (Ct) method and normalized to respective GAPDH controls.

### Engineering Dox-inducible plasmids and stable cell lines

The full-length cDNA of *TLK2* was purchased from Origene (Catalogue #: SC115810), and the open reading frame (ORF) was subcloned into an inducible lentiviral pTINDLE vector provided by Dr Xuewen Pan. This vector contains an inducible promoter (pTRE-tight) and a transactivator (rtTA3) in a lentiviral backbone. We also engineered the ORF of Yellow Fluorescent Protein (YFP) into the pTINDLE vector as a control. TLK2 shRNA (5′-CCCAGAATAGTTAAGCTGT-3′) and non-silencing controls (5′-ATCTCGCTTGGGCGAGAGTAAG-3′) were purchased from Open Biosystems. The shRNA was engineered into another inducible lentiviral pINDUCER vector^50^. After lentivirus packaging, cells were infected by lentivirus containing doxycycline (Dox) inducible plasmid, adding 8 μg ml^−1^ polybrene. Stable cell lines expressing shTLK2 were established by sorting GFP-positive cells using a flow cytometric cell sorter, FACSAria (BD Biosciences). The stable lines expressing the *TLK2* ORF were selected by treating with Geneticin (Invitrogen). 2 μg ml^−1^ of Dox (Sigma-Aldrich) was used for shTLK2 induction and 0, 50, 100 or 200 ng ml^−1^ of Dox was used to express the *TLK2* ORF. For inducible overexpression systems, TLK2 expression was induced for two weeks before the stable lines were subjected to phenotypic assays.

### Clonogenic assay

Cells (3,000–5,000) were seeded in 6-well plates and incubated for 14–21 days. For the Dox-inducible TLK2 overexpression model, 100 ng ml^−1^ of Dox was added for 2 weeks before the clonogenic assay. For the Dox-inducible TLK2 knockdown model, 0.5 μg ml^−1^ of Dox was added for 2 days before the clonogenic assay. The colonies were stained with 0.5% crystal violet and 50% methanol and were counted by a GelCount colony counter (Oxford Optronix).

### Soft-agar colony formation assay

Cells (3,000–5,000) were suspended in growth medium containing 0.35% SeaPlaque Agarose (Lonza), and plated on 0.5% base agar in 6-well plates. Then cells were incubated at 37 °C in 5% CO_2_ for 14–21 days, and colonies were counted using GelCount (Oxford Optronix Ltd.). For the Dox-inducible TLK2 overexpression model, 100 ng ml^−1^ of Dox was added for 2 weeks before the clonogenic assay. For the Dox-inducible TLK2 knockdown model, 0.5 μg ml^−1^ of Dox was added for 2 days before the clonogenic assay.

### Transwell migration and invasion assay

Boyden chambers were used for transwell migration and invasion assays. Cells were serum-starved for 24 h and 5 × 10^4^∼3 × 10^5^ cells were seeded with serum-free medium into the top of the transwell inserts with 8 μm pore size for the migration assay, or into the top of the transwell coated with matrigel (BD Biosciences) for the invasion assay. In the bottom chamber, regular medium containing serum was added. To facilitate the migration of MCF7, MDAMB361, or T47D cells, NIH3T3 cells were seeded in the bottom chamber as a chemo-attractant. For the Dox-inducible TLK2 overexpression model, 0, 50, 100, or 200 ng ml^−1^ of Dox was administered for 2 weeks before the migration and invasion assay. To verify the dependence of migration and invasion properties on TLK2 expression, Dox was withdrawn for 4 days following 2 weeks of Dox treatment to deplete the excess TLK2 protein. To observe the effect of SRC, EGFR or FAK inhibition on TLK2-driven cell motility, 20 nM of siRNA targeting EGFR and FAK or 20–40 nM of SRC siRNA (Dharmacon) were transfected for 3 days before perform the transwell migration assay. After 48–72 h, the inserts were fixed in 4% formaldehyde and stained with hematoxylin and eosin. The migrated and invaded cells were counted by GelCount colony counter (Oxford Optronix Ltd.).

### Immunoprecipitation assay

Rabbit monoclonal antibody against SRC (Cell Signalling #2123) was conjugated with protein A/G-sepharose beads (Santa Cruz). 500 μg of fresh protein lysates from cells were immunoprecipitated for overnight at 4 °C with constant rotation. After washing three times with extraction buffer, proteins that co-immunoprecipitated were analysed by western blot as described previously. Protein lysate (30–50 μg) was loaded as a control input.

### Reverse phase protein array analysis

Reverse phase protein array assays were carried out as described previously with minor modifications^51^. Protein lysates were prepared from cultured cells or tissue samples with modified Tissue Protein Extraction Reagent (TPER) (Pierce) and a cocktail of protease and phosphatase inhibitors (Roche Life Science). The lysates were diluted into 0.5 mg ml^−1^ of total protein in SDS sample buffer and denatured on the same day. The Aushon 2470 Arrayer (Aushon BioSystems) with a 40 pin (185 μm) configuration was used to spot samples and control lysates onto nitrocellulose-coated slides (Grace Bio-labs) using an array format of 960 lysates/slide (2880 spots/slide). The slides were processed as described^51^ and probed with a set of 200 antibodies against total and phosphoprotein proteins using an automated slide stainer Autolink 48 (Dako). Each slide was incubated with one specific primary antibody and negative control slide was incubated with antibody diluent instead of primary antibody. Primary antibody binding was detected using a biotinylated secondary antibody followed by streptavidin-conjugated IRDye680 fluorophore (LI-COR Biosciences). Total protein content of each spotted lysate was assessed by fluorescent staining with Sypro Ruby Protein Blot Stain according to the manufacturer's instructions (Molecular Probes). Flurosecent-labeled slides were scanned on a GenePix AL4200 scanner, and the images were analysed with GenePix Pro 7.0 (Molecular Devices). Total fluorescence signal intensities of each spot were obtained after subtraction of the local background signal for each slide and were then normalized for variation in total protein, background and non-specific labelling using a group-based normalization method as described^51^. For each spot on the array, the-background-subtracted foreground signal intensity was subtracted by the corresponding signal intensity of the negative control slide (omission of primary antibody) and then normalized to the corresponding signal intensity of total protein for that spot. The median of the triplicate experimental values (normalized signal intensity) is taken for each sample for subsequent statistical analysis. *T*-tests are performed using Perl module ‘Statistics::T-Test'.

### FACS analysis of cell cycle and apoptosis

For cell cycle analysis, cells were fixed in 70% EtOH and then stained with propidium iodide (Sigma-Aldrich). For cell apoptosis analysis, cells were stained using the Annexin V-FITC apoptosis detection kit following manufacturer's protocols (Abcam). Cells were analysed using FACSCantoll cell analyzer (BD Biosciences) and Flowjo software.

### Double thymidine or nocodazole block and BrdU incorporation

MCF7 cells were blocked at the G1/S border or in mitosis using the following protocols. MCF7 cells were blocked with 2.5 mM Thy for 18 h, released for 9 h after washing with PBS for three times, and then blocked again with 2.5 mM Thy for 17 h. To synchronize MCF7 cells in mitosis, cells were incubated with 200 nM of nocodazole for 15 h. After shaking off the cells, floating cells were collected to obtain the mitotic cell population and then cells were released by washing with PBS for three times. To precisely determine the S-phase cell population, 10 μM of BrdU was added for 1.5 h before cell collection.

### Fluorescence *in situ* hybridization assay

For FISH analysis, exponentially growing cells were treated with Colcemid (0.04 μg ml^−1^) for one hour at 37 °C followed by hypotonic treatment (0.075 M KCl) for 20 min at room temperature. Cells were fixed in a methanol and acetic acid (3:1 by volume) mixture for 15 min, and washed three times in the fixative. Slides were prepared by dropping the cell suspension on wet slides and air drying. FISH was performed on these slides using TLK2 (Red 5-Rox dUTP) and centromere 17 (Green 5-Fluorescein dUTR) probes from Empire Genomics, Buffalo, NY. Probe (10 μl) was placed on each slide, covered with cover glass and sealed with rubber cement. The slides and the probe were co-denatured at 72 °C for 3 min in ThermoBrite hybridzer, and then incubated at 37 °C in a humid chamber overnight. The slides were washed in 0.4XSSC/0.3% Tween20 at 72 °C for 2 min and in 2 × SSC at room temp for 2 min. The slides were counterstained with DAPI, and the images were captured using Nikon 80i microscope equipped with a cooled-charge coupled devices (CCD) camera. A total of 50 interphase nuclei were analysed to determine the amplification status.

### *In vivo* xenograft experiments

All animal experiments have been approved by the BCM Institutional Animal Care and Use Committee. MCF7 cells (7.5 × 10^6^) with Dox-dependent expression of shTLK2 were injected bilaterally to 4–6 week old female athymic nude mice (Harlan Sprague-Dawley) supplemented with 17β-estradiol pellets. Xenograft tumours of the MCF7 models were successfully engrafted in 32 mice which were randomized into ±doxycycline (Dox) with or without tamoxifen treatment (8 mice per group). Briefly, when tumours reached 200 mm^3^, tamoxifen (25 μg kg^−1^ body weight, 5 days weekly) was injected subcutaneously and 0.2 mg ml^−1^ Dox were administered with drinking water. The growth of the xenograft tumours was monitored twice per week and tumour volume was measured using the formula; tumour volume=1/2(length × width^2^). Mice were sacrificed and tumours were harvested when they reached 1,500 mm^3^, or at the end of the experiment. To observe relative long-term therapeutic effects, mice were monitored for up to 100 days depending on the duration of tumour control. An additional 5 mice per group were included for the analyses of biomarkers, for which tumours were harvested after two weeks of treatments.

### Statistical analysis

The results of the *in vitro* experiments were analysed by Student's *t*-tests, and all data are shown as mean±s.d.. For the *in vivo* study, the generalized Wilcoxon test was used for progression-free survival analysis and ANOVA was applied for tumour volume analysis in different treatment groups.

### Data availability

The TCGA RNAseq and copy-number data sets used in this study are available from TCGA portal (http://cancergenome.nih.gov/). The Metabric copy-number and gene expression data sets are available from European Genome-Phenome Archive (EGA: http://www.ebi.ac.uk/ega/), under accession number EGAS00000000083. Copy-number (Affymetrix SNP 6.0 array) and gene expression data (Affymetrix GeneChip Human Exon 1.0 ST Array) for breast cancer cell lines are available from EGA under accession number EGAS00000000059 and ArrayExpress (http://www.ebi.ac.uk/arrayexpress/) under accession number E-MTAB-181 respectively^21^. The RPPA data for the MCF7-TLK2 knockdown models are available in Supplementary Data 1. All other data is included in the Article or Supplementary Files or available from the authors upon request.

## Additional information

**How to cite this article:** Kim, J. A. *et al*. Comprehensive functional analysis of the *tousled-like kinase 2* frequently amplified in aggressive luminal breast cancers. *Nat. Commun.* **7,** 12991 doi: 10.1038/ncomms12991 (2016).

## Supplementary Material

Supplementary Information Supplementary Figures 1-13, Supplementary Tables 1-3 and Supplementary Reference.

Supplementary Data 1 Reverse Phase Protein Array (RPPA) data
