# Supplementary material for: Comprehensive functional analysis of the tousled-like kinase 2 frequently amplified in aggressive luminal breast cancers
Source: Nat Commun. 2016 Oct 3;7:12991. doi: 10.1038/ncomms12991 (PMC5064015; doi:10.1038/ncomms12991)
Supplement: Supplementary Information — Supplementary Figures 1-13, Supplementary Tables 1-3 and Supplementary Reference. [file ncomms12991-s1.pdf]

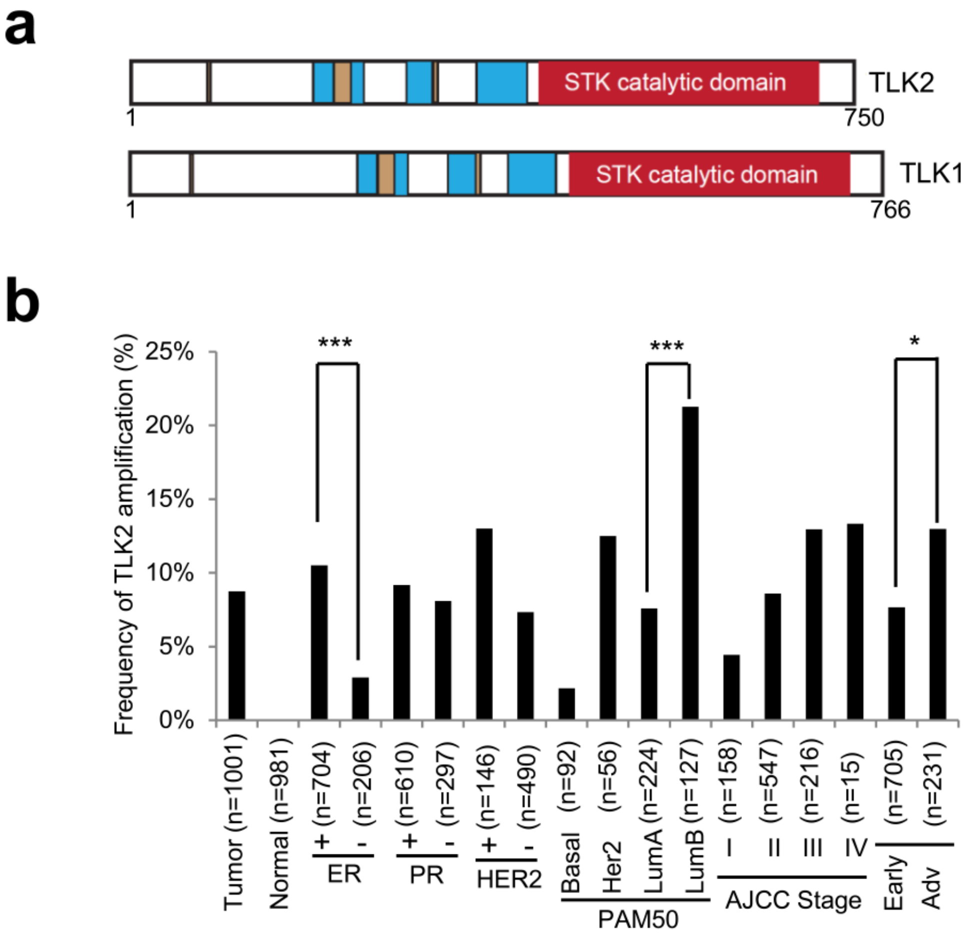

Supplementary Figure 1. The structure of tousled-like proteins and the frequencies of *TLK2* amplifications in different breast cancer clinical subtypes. (**a**) The domain architecture of TLK2 protein in comparison with TLK1 protein. The predicted coiled-coil regions are shown in blue boxes, and putative nuclear localization sequences are shown in light brown boxes. The figure is adapted with permission from Sillje et al. EMBO J. 1999, 18:5691 [ref.1]. (**b**) The frequencies of genomic amplifications were assessed based on Affymetrix SNP 6.0 copy number data for 1001 invasive breast tumors from TCGA. Normal, available paired normal blood or adjacent normal breast tissues. Samples with relative *TLK2* copy number >0.7 are considered as positive. *TLK2* is more frequently amplified in ER+ than ER- breast cancers, and this event is most frequent in the Luminal B subtype. Early, early stage breast cancer (Stage I-II); Adv, advanced stage breast cancer (stage III-IV). *, p<0.05; ***, p<0.001 (Fisher's exact test).

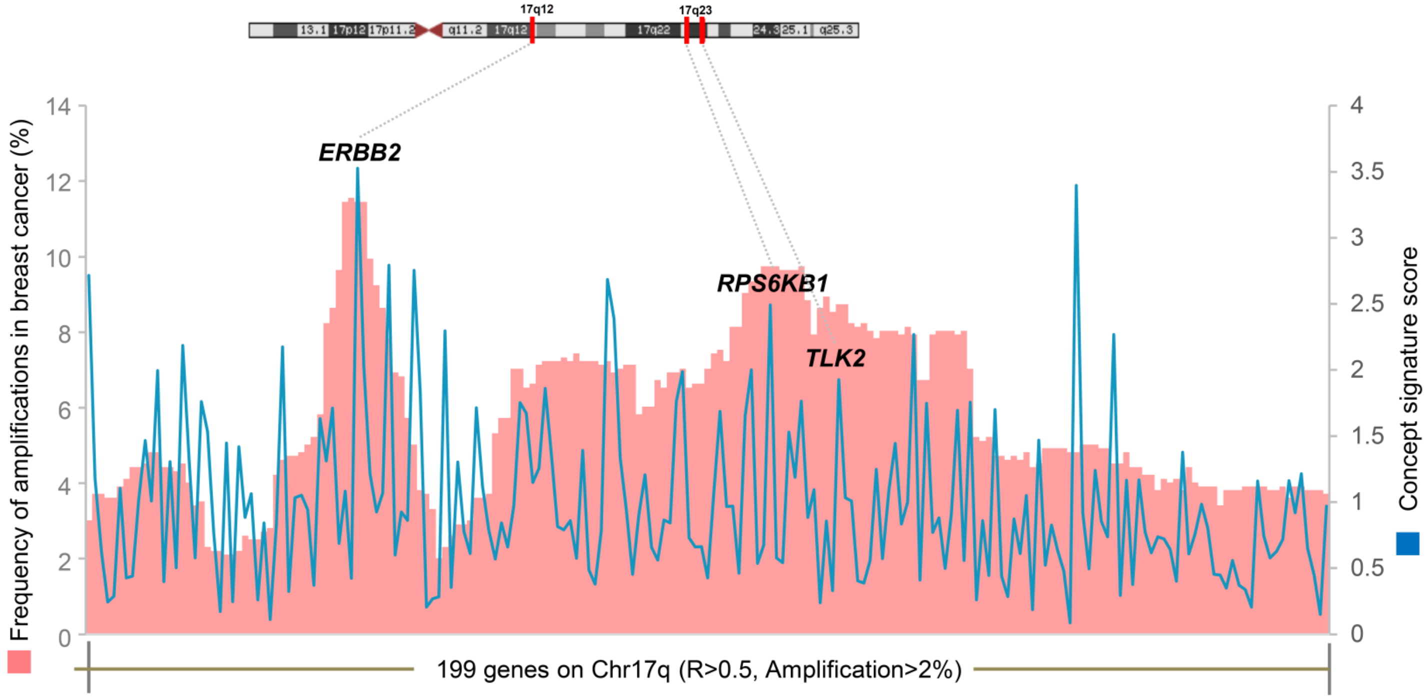

Supplementary Figure 2. Frequent gene amplifications in Chr17q with significantly correlated gene expressions. Chr17q genes amplified in >2% of breast cancers as well as having Spearman's correlation coefficient R>0.5 are shown in the chart. The concept signature scores for these genes are shown in the blue line chart. The three lead amplified kinase targets (*ERBB2*, *RPS6KB1*, and *TLK2*) nominated by ConSig-Amp analysis are shown in the chart. All three targets locate at the peaks of both genomic amplifications and ConSig scores. This coincidence provided integrated evidence about their functional importance in breast cancer. TLK2 locates in a small peak region of genomic amplifications close to the *RPSKB1* amplicon. This figure is based on the copy number data and RNAseq expression data from TCGA.

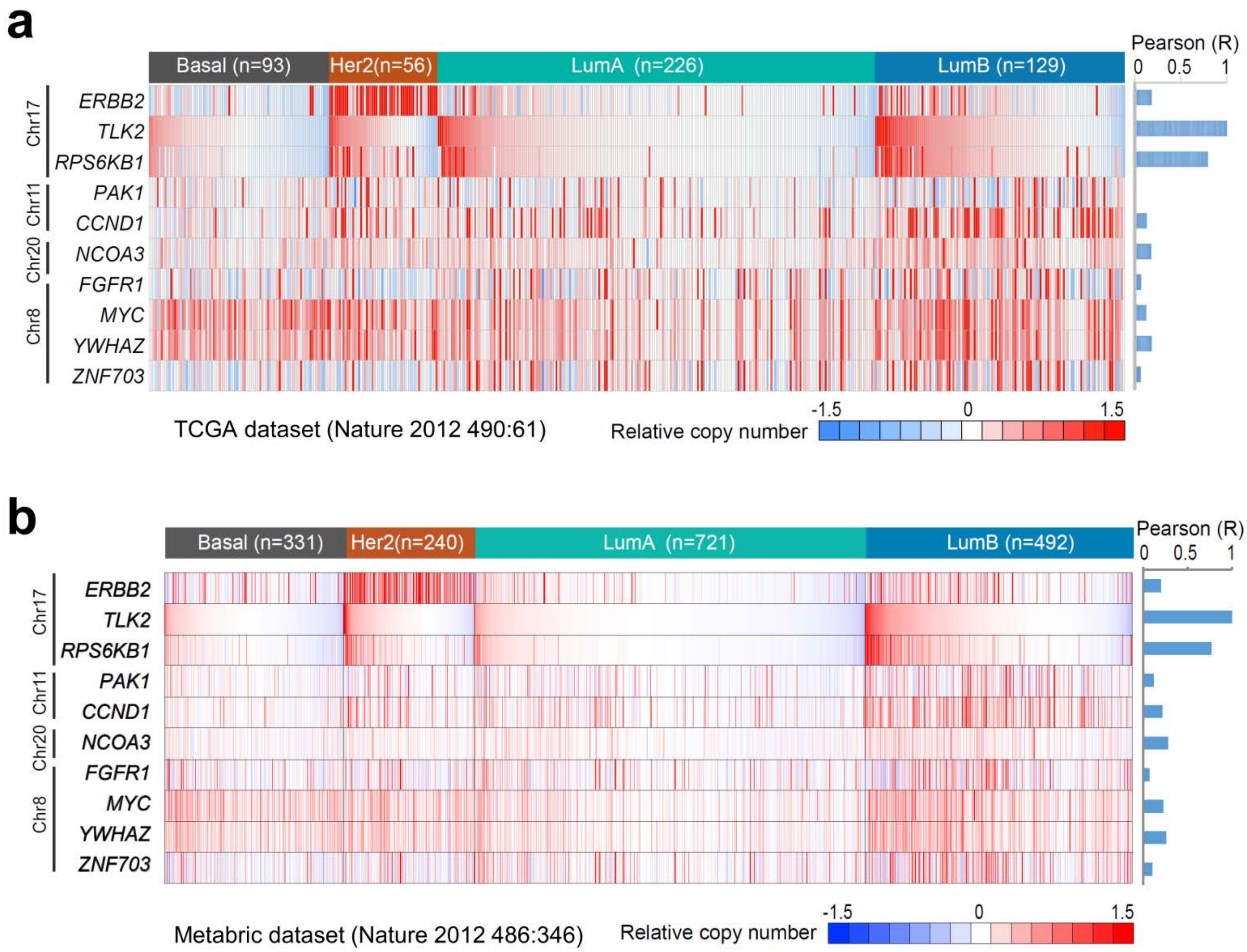

Supplementary Figure 3. Correlation of *TLK2* copy numbers with known amplified oncogenes in breast cancer based on (a) TCGA dataset[12] and (b) Metabric dataset[18]. The copy number data of *TLK2* and known amplified oncogenes including *ERBB2*, *RPS6KB1*, *PAK1*, *CCND1*, *NCOA3*, *FGFR1*, *MYC*, *YWHAZ, and ZNF703* are shown in the heat-maps. The Pearson correlation coefficients (R) of copy number data between known amplified oncogenes and *TLK2* are shown in the right bar charts (R=1 for *TLK2* itself). Here the Pearson's correlation statistic is used as the copy number data between co-amplified genes are expected to be linearly correlated. The samples are sorted based on PAM50 clinical subtypes and then sorted based on *TLK2* copy number. As shown in the figure, *TLK2* copy number does not correlate with most known oncogene amplifications, except *RPSKB1*.

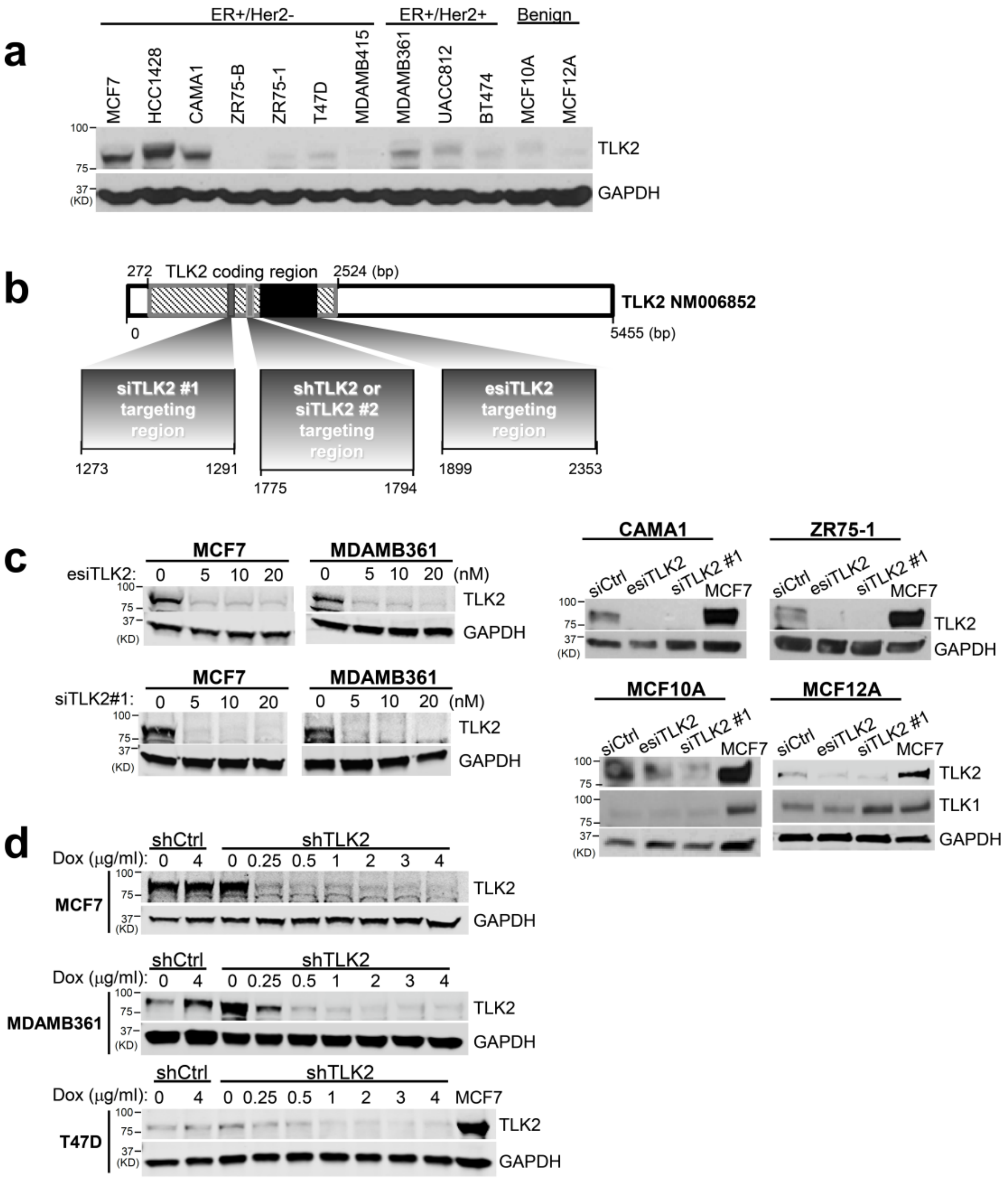

Supplementary Figure 4. TLK2 protein expression in ER+ breast cancer cell lines and validation of TLK2 silencing by esiTLK2, siTLK2, and shTLK2. (**a**) TLK2 protein expression in ER+ breast cancer cell lines and benign breast epithelial cells. (**b**) Schematic showing the target regions of esiTLK2, siTLK2, and shTLK2. (**c**) Western blot validation of TLK2 silencing in MCF7, MDAMB361, CAMA1, ZR75-1, MCF12A, and MCF10A cells treated with 10 nM siRNA#1 or esiRNA. MCF7 was used as positive control for the CAMA1, ZR75-1, MCF10A, and MCF12A cells. (**d**) Western blot validation of TLK2 silencing by TLK2 shRNA inducibly expressed for 72hr in MCF7, MDAMB361, or T47D cells. The parental MCF7 cell line was used as positive control for the T47D-shTLK2 model. KD, knockdown.

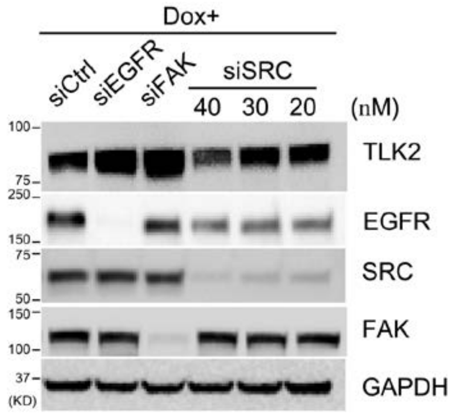

Supplementary Figure 5. Western blots showing effective silencing of EGFR, SRC, and FAK by siRNAs in T47D overexpressing TLK2. This figure corresponds to the experiment shown in Figure 3f. TLK2 expression was induced in engineered T47D cells by treating 200 ng/ml Dox for 2 weeks. The siRNAs against EGFR, SRC, or FAK were transfected for 3 days. For siRNA transfection, 40 nM of siCtrl, 20 nM of siEGFR or siFAK and indicated concentration of siSRC were used. Dox, doxycyclin.

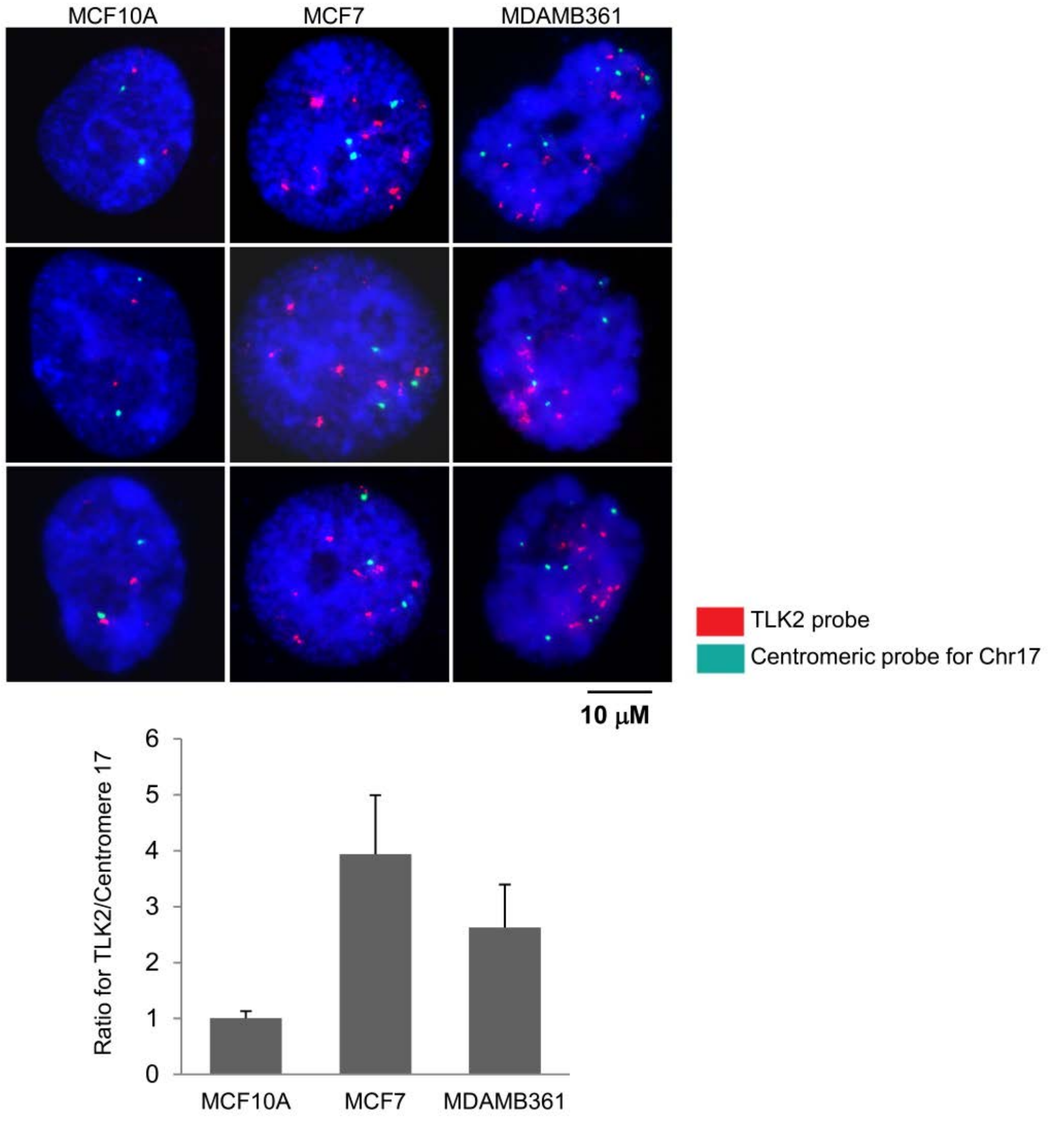

Supplementary Figure 6. Fluorescence in situ hybridization (FISH) on *TLK2*-amplified MCF7 and MDAMB361 breast cancer cells compared to MCF10A benign breast epithelial cells. The FISH assay is developed using a probe specific to the *TLK2* locus (TLK2 Red 5-Rox dUTP) and a probe for centromere 17 (CEN17 Green 5-Fluorescein dUTR) obtained from Empire Genomics. The upper panel shows the representative FISH images, and the lower panel shows the quantitative ratios of TLK2 signals against centromere 17 signals (fifty cells were counted for each cell line). Normally, two copies of the TLK2 signals are found in MCF10A benign epithelial cells, whereas multiple copies of the TLK2 signals were observed in MCF7 and MDAMB361 cells. The average TLK2:CEN17 ratios for MCF7 and MDAMB361 are 3.94 and 2.63 respectively, indicating genome amplifications. As a reference, the cut-off for *Her2* amplification in FISH is HER2:CEN17 ratio ≥2.0 (Garcia-Caballero T, et al. Histopathology 56:472-480). Error bars represent the standard deviation of TLK2:CEN 17 ratios of fifty cells per condition.

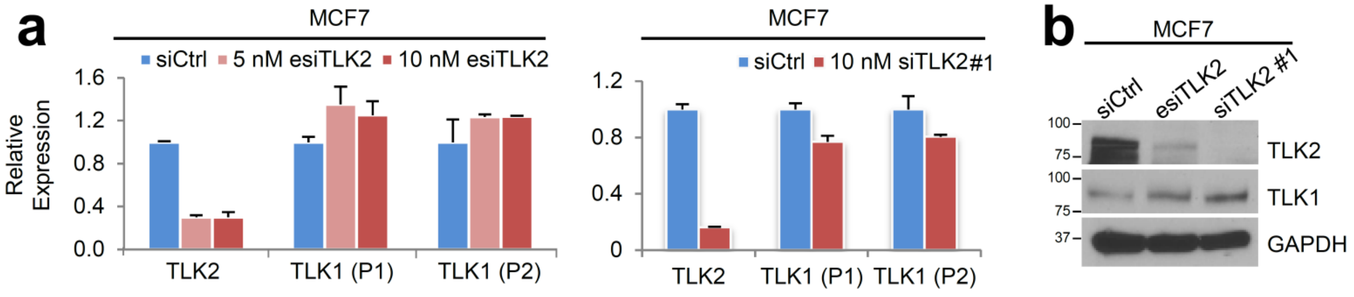

Supplementary Figure 7. Verifying the specificity of TLK2 siRNAs against TLK2 but not TLK1. (**a**) Quantitative PCR (qPCR) verifying the specific knockdown of TLK2 in MCF7 by esiTLK2 or siTLK2#1 without significantly affecting TLK1 expression. One primer pair was used for TLK2 qPCR, and two primer pairs P1 and P2 were used for TLK1 qPCR. Error bars represent the standard deviation of three replicate measurements per condition. (**b**) Western blot showing TLK2 specific knockdown by esiTLK2 or siTLK2 #1 in MCF7 cells.

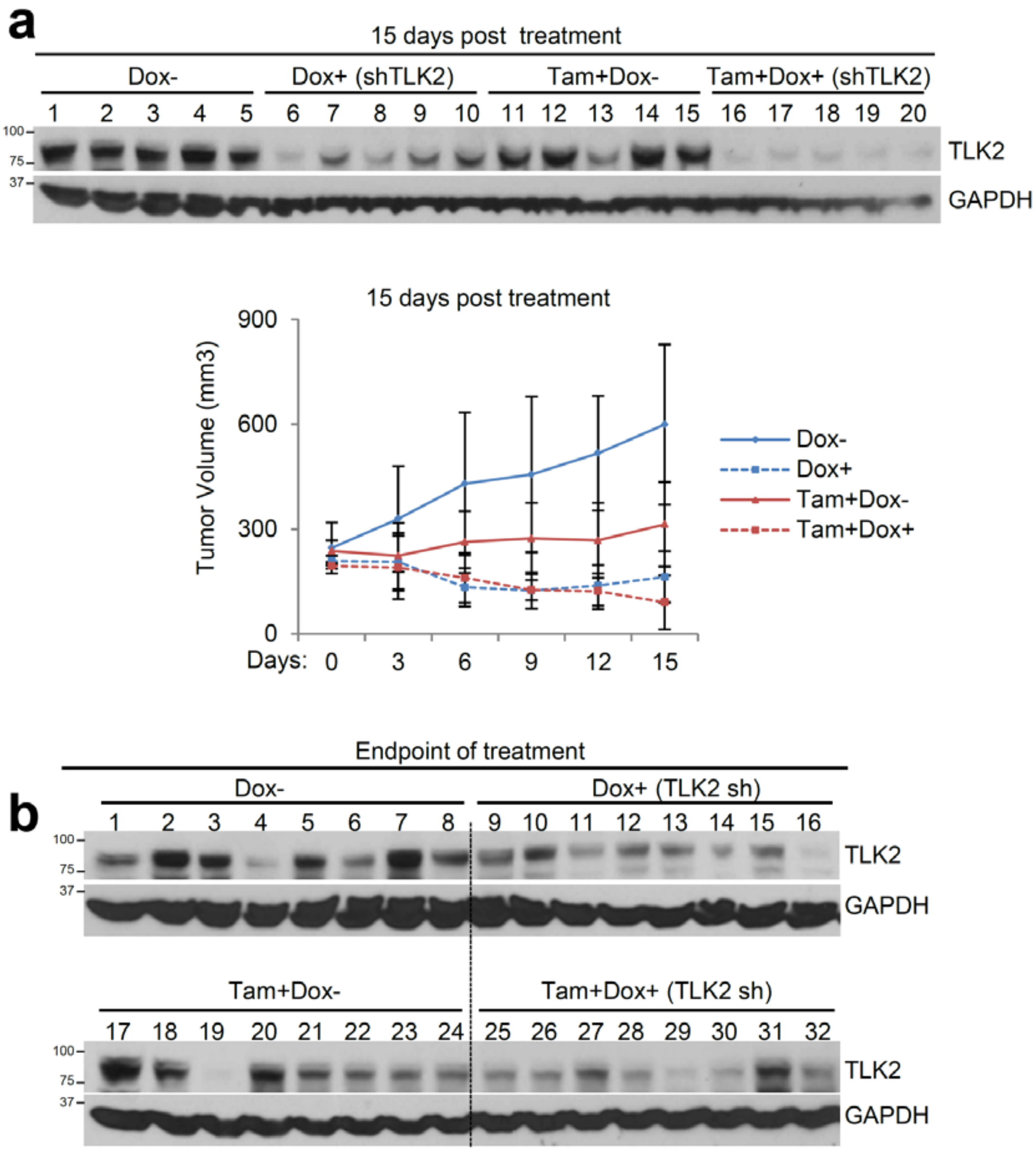

Supplementary Figure 8. Verifying the effectiveness of inducible TLK2 inhibition *in vivo* by western blot analysis of tumors harvested 2 weeks after treatment or at the endpoint of treatment. (**a**) The upper panel shows TLK2 protein level from the tumors collected 2 weeks post treatment. The lower panel shows the tumor growth curve of each treatment group until 2 weeks of treatment before the tumors were harvested. Five mice were included in each group. Error bars represent the standard deviation of tumor volumes of five mice measurements per condition. (**b**) The TLK2 protein levels from tumors collected at the endpoint of treatment. Eight mice were included in each group. The quantitative results of western blots are shown in Fig. 5c. Dox, doxycycline.

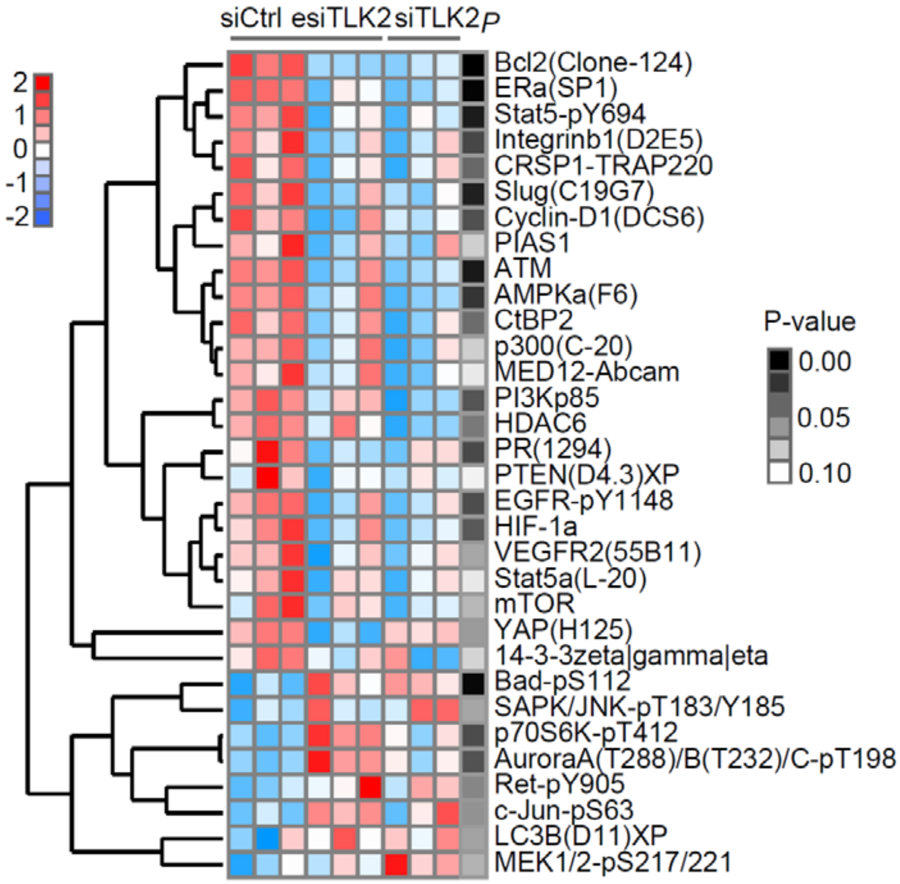

Supplementary Figure 9. The heat-map of proteins that are altered after TLK2 knockdown in MCF7 cells (p<0.1). For RPPA profiling, experiments were biologically repeated three times. *P*-values were calculated based on *t*-test. For data visualization, protein signals are normalized by subtracting the mean and dividing with standard deviation of each protein. The proteins are clustered by the complete linkage method of hierarchical clustering.

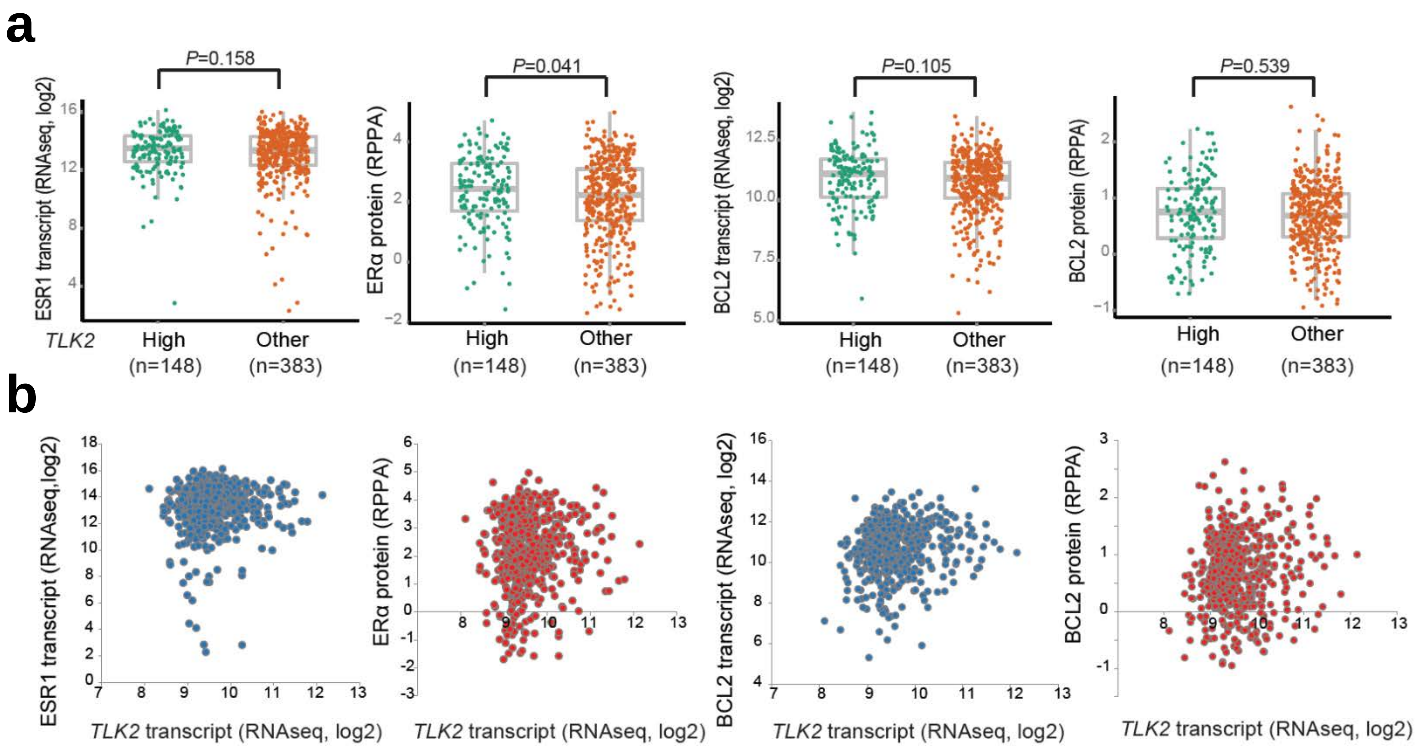

Supplementary Figure 10. Correlation of ESR1 and BCL2 transcript or protein expressions with TLK2 transcript expression in 531 ER-positive breast tumors profiled by TCGA. (**a**) Box plot comparing ESR1 and BCL2 protein expression (based on RPPA data) or transcript expression (based on RNAseq data) in ER-positive breast tumors profiled by TCGA with or without TLK2 overexpression. P-values are based on *t*-test. (**b**) Dot plot showing the correlation of ESR1 and BCL2 transcript or protein expression with TLK2 transcript expression in TCGA ER-positive breast tumors. The TCGA RPPA data used in this figure are based on replicate-base normalization and are downloaded from the Cancer Genome Browser (https://genome-cancer.ucsc.edu).

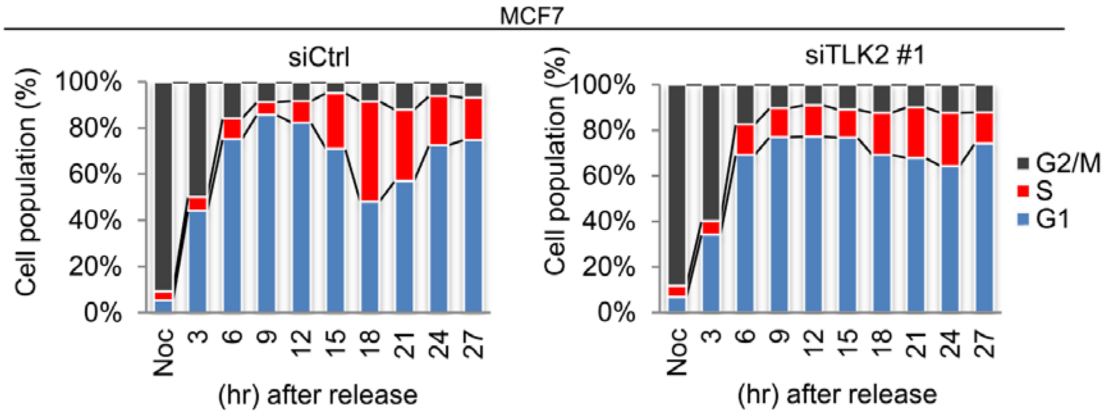

Supplementary Figure 11. Alterations of cell cycle profile after TLK2 silencing using TLK2 siRNA#1. MCF7 cells were synchronized via nocodazole-induced mitotic block. "Noc" indicates the MCF7 cells synchronized at mitosis by nocodazole block. The cell cycle profile is based on the DNA content quantitated by flow cytometry.

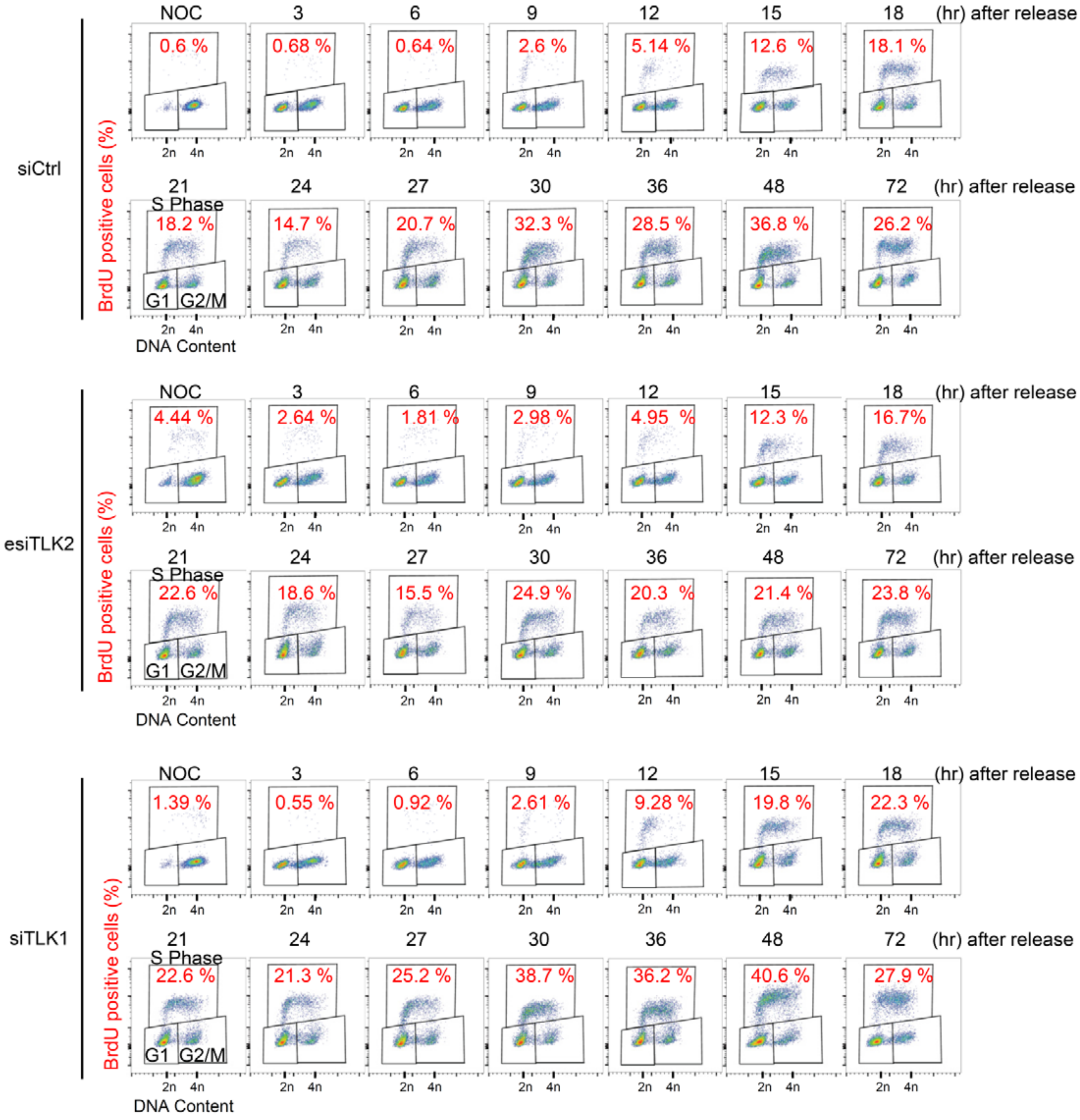

Supplementary Figure 12. Alterations of cell cycle progression after TLK2 or TLK1 silencing determined based on DNA content and BrdU incorporation. Cell cycle profile was analyzed after TLK2 or TLK1 knockdown by transfecting TLK2 esiRNA or TLK1 siRNA in MCF7 cells synchronized via nocodazole-induced mitotic block. Cells were released from mitosis as described in METHODS. To precisely determine S-phase cell population, 10 mM BrdU was added for 1.5 h prior to cell collection. This data correspond to the cell cycle profile figure shown in Fig.7c, upper panel. "Noc" indicates MCF7 cells synchronized at mitosis by nocodazole block.

Figure 2b

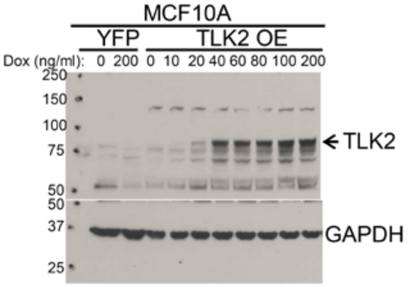

Figure 3a

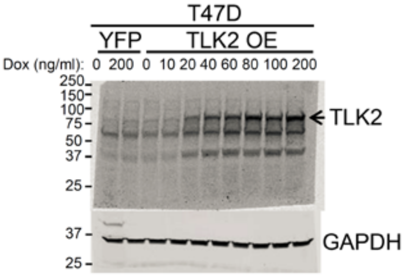

Figure 4e

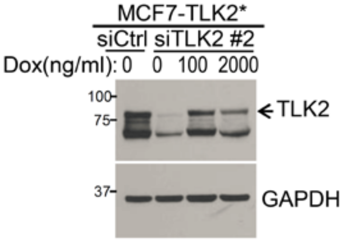

Figure 3e

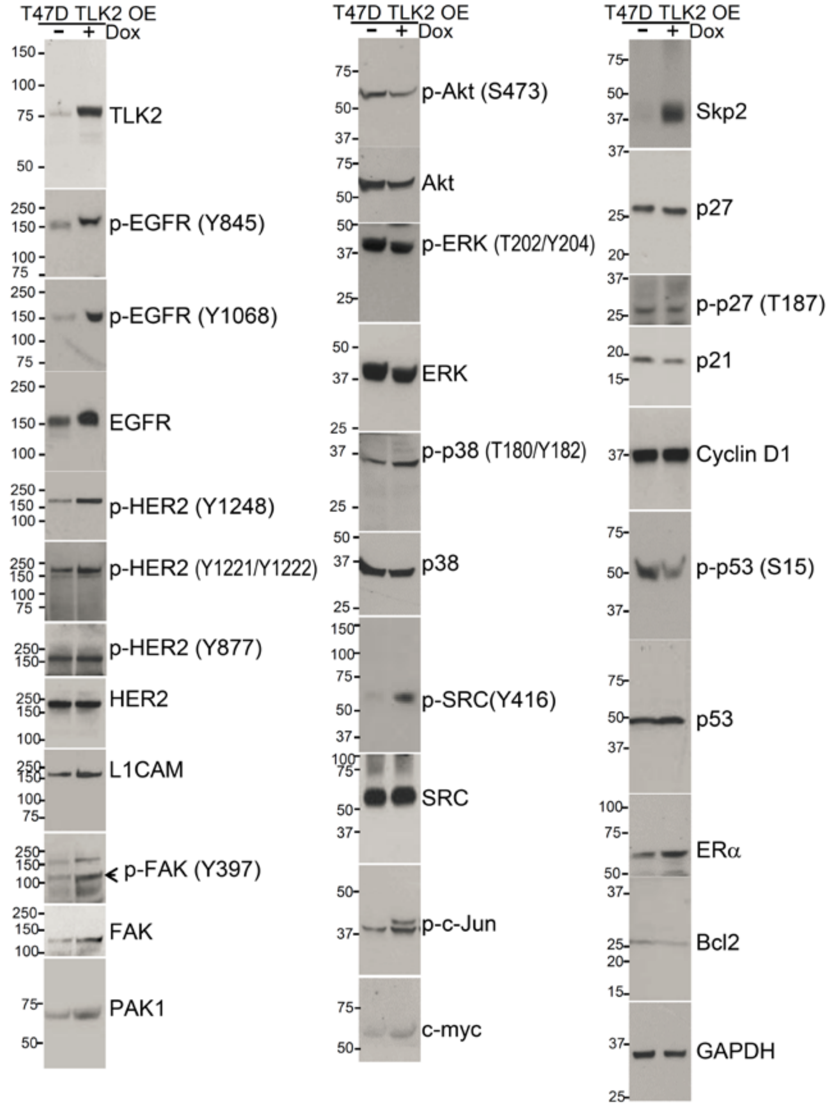

Figure 6c

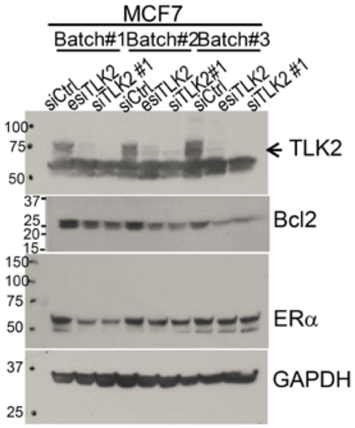

Supplementary Figure 13. Uncropped western blot images for main figures.

Figure 7d

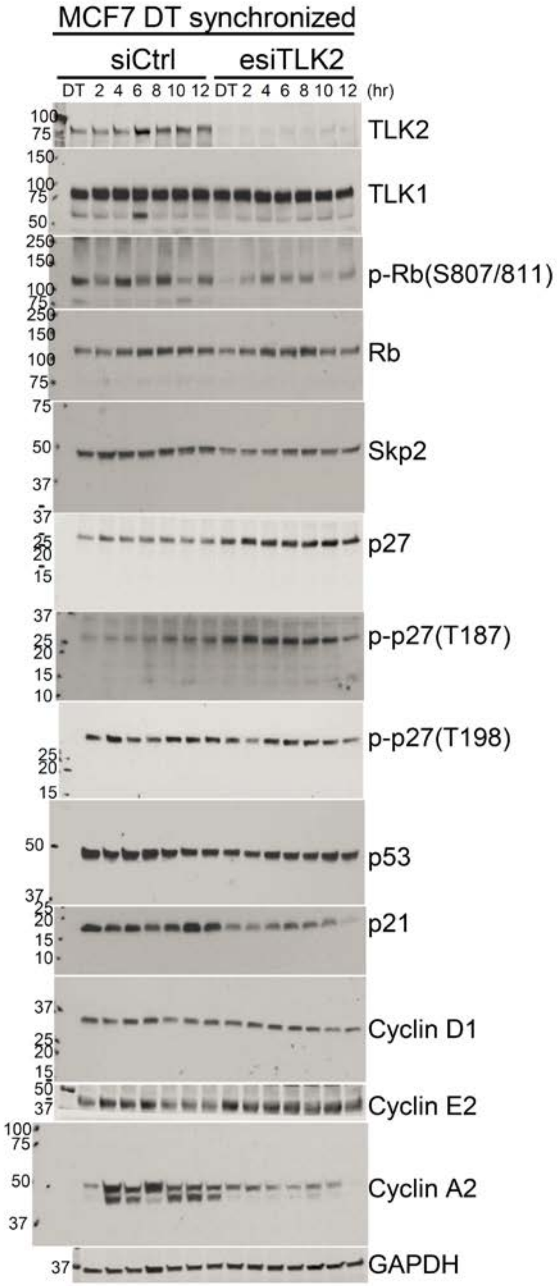

Figure 8b

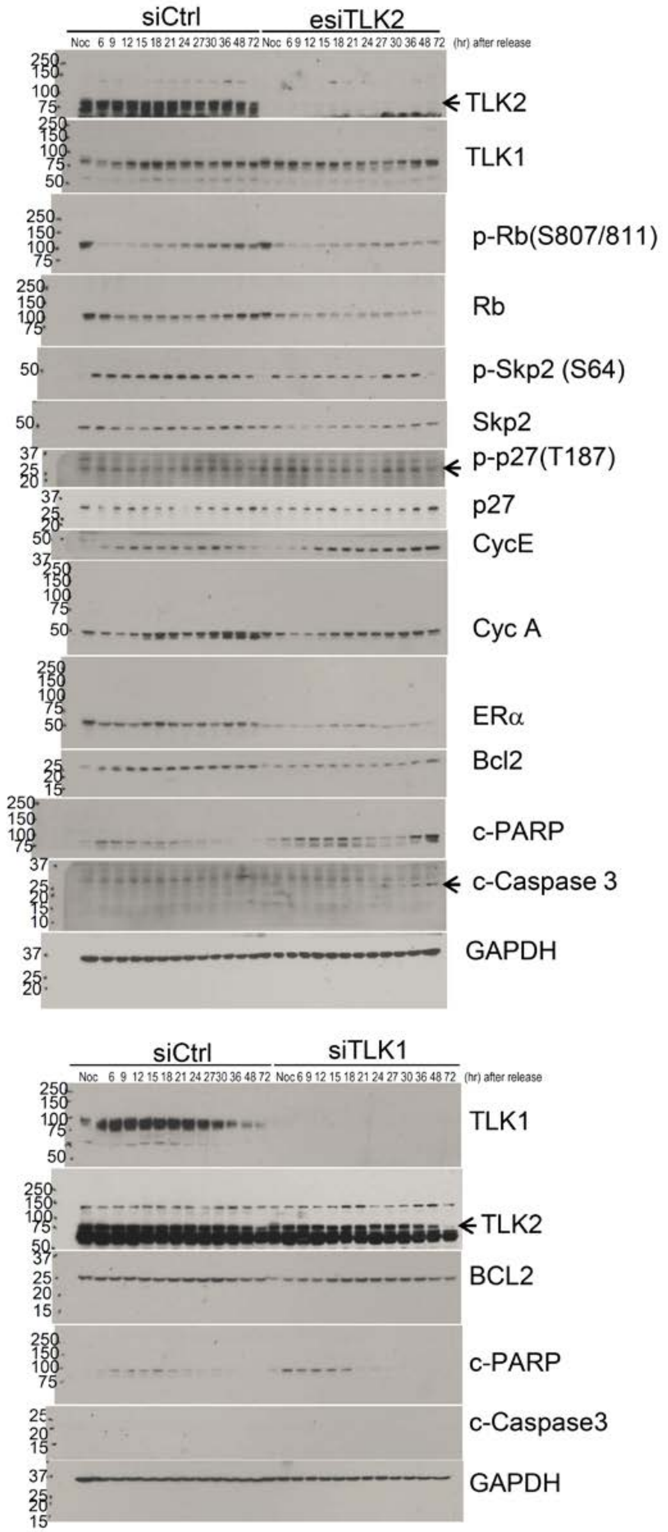

Supplementary Figure 13-continued. Uncropped western blot images for main figures.

Gene Symbol	Chr	Druggable	Kinase	ConSig	Spearman (R)	ConSig-amp score	Amp% in ER+ (n=704)	Amp% in LumA (n=224)	Amp% in LumB (n=127)
ERBB2	chr17	Y	Y	3.53	0.71	2.49	10.2	4.0	11.0
PTK2	chr8	Y	Y	2.87	0.85	2.45	16.2	10.7	28.3
RPS6KB1	chr17	Y	Y	2.49	0.78	1.94	11.9	7.6	20.5
GRB2	chr17	Y		3.40	0.54	1.84	5.4	3.6	9.4
TLK2	**chr17**	**Y**	**Y**	**1.93**	**0.81**	**1.55**	**10.5**	**7.6**	**21.3**
WHSC1L1	chr8	Y		1.83	0.80	1.46	13.2	12.9	20.5
THRA	chr17	Y		2.79	0.51	1.43	7.1	3.6	7.9
IKBKB	chr8	Y	Y	2.51	0.57	1.42	10.5	8.9	17.3
PAK1	chr11	Y	Y	2.50	0.57	1.42	7.7	8.5	13.4
DDX5	chr17	Y		2.27	0.62	1.41	8.9	6.7	15.7
PRKDC	chr8	Y	Y	2.20	0.62	1.37	9.9	9.4	18.1
PTPN1	chr20	Y		2.39	0.57	1.35	7.1	4.9	11.0
PARP1	chr1	Y		1.99	0.66	1.31	10.2	11.6	11.8
SPAG9	chr17	Y		2.39	0.54	1.29	8.2	7.1	12.6
MED1	chr17	Y		1.71	0.70	1.19	8.2	3.6	11.8
KAT6A	chr8	Y		1.75	0.68	1.18	11.2	10.7	16.5
MTMR4	chr17	Y		1.69	0.70	1.18	9.2	5.8	17.3
PPM1D	chr17	Y		1.76	0.66	1.17	11.9	7.6	20.5
DHX40	chr17	Y		1.65	0.69	1.14	11.2	6.3	21.3
PIP5K1A	chr1	Y	Y	1.53	0.74	1.13	9.2	12.1	11.0
STK3	chr8	Y	Y	1.57	0.69	1.08	17.2	12.5	29.1
NDUFS2	chr1	Y		1.43	0.74	1.06	10.7	11.6	14.2
RB1CC1	chr8	Y	Y	1.58	0.67	1.06	10.5	8.0	19.7
PPP1R9B	chr17	Y		1.86	0.55	1.03	8.1	6.3	11.8
GNAS	chr20	Y		2.04	0.50	1.02	7.8	4.9	14.2
PRKAR1A	chr17	Y	Y	1.76	0.56	0.98	7.8	5.4	15.7
TPR	chr1	Y		1.68	0.58	0.98	8.9	10.7	11.0
USP32	chr17	Y		1.53	0.63	0.97	11.8	7.1	20.5
SDHC	chr1	Y		1.26	0.74	0.94	10.5	11.6	14.2
ADSS	chr1	Y		1.37	0.67	0.92	10.4	11.6	11.0
PPP1CA	chr11	Y		1.61	0.57	0.92	7.2	4.5	15.0
ATAD2	chr8	Y		1.32	0.69	0.91	19.0	12.9	33.1
SQLE	chr8	Y		1.29	0.70	0.90	19.6	13.8	34.6
FH	chr1	Y		1.32	0.68	0.90	10.4	11.6	11.0
DDX42	chr17	Y		1.25	0.71	0.89	9.1	6.3	16.5
XYLT2	chr17	Y		1.36	0.63	0.86	8.2	7.1	11.0
AURKA	chr20	Y	Y	1.50	0.55	0.83	7.0	4.0	13.4
PROSC	chr8	Y		0.97	0.84	0.82	14.5	14.3	24.4
ATP6V1C1	chr8	Y		1.08	0.76	0.81	18.2	13.8	29.9
MCM4	chr8	Y		1.33	0.61	0.81	9.9	9.4	18.1
FNTA	chr8	Y		1.04	0.77	0.80	8.0	8.0	13.4
NME1	chr17	Y	Y	1.33	0.60	0.80	8.4	6.7	14.2

PPP2R5A	chr1	Y		1.28	0.61	0.78	11.4	12.9	11.8
SETDB1	chr1	Y		0.99	0.79	0.78	9.9	12.1	12.6
PDK2	chr17	Y	Y	1.26	0.60	0.76	8.0	6.3	11.8
EPRS	chr1	Y		1.42	0.53	0.75	10.4	12.1	10.2
PIP4K2B	chr17	Y	Y	0.94	0.77	0.72	5.3	2.2	7.9
CLK2	chr1	Y	Y	1.10	0.66	0.72	9.9	12.1	12.6
CCT3	chr1	Y		1.29	0.56	0.72	8.4	12.1	7.1
ARF1	chr1	Y		1.11	0.65	0.72	10.2	12.1	11.8

Supplementary Table 1. Top 50 druggable candidate oncogenes targeted by frequent genomic amplifications in breast cancers. Genes are sort by the ConSig-amp scores. The criteria of nomination: a) amplified in >5% of ER+ breast cancers; b) gene expression correlates with copy number (R>0.5); c) druggable. The candidates are then prioritized based on ConSig score and the Spearman's correlation (R) between gene expression and copy number (data shown here are part of Fig. 1A). ConSig-amp score = ConSig× Spearman (R).

**TLK2 RT-PCR primers**	
**TLK2_F1**	GACCGCTTGAGACTGGGCCACT
**TLK2_R2**	AATGAGGCTCCGTGTCGGACAG
**TLK1 RT-PCR primers**	
**TLK1_F1**	TAAGCAGTGAAGCCAAGGCA
**TLK1_R1**	GAAGAAGGGGGTGTAGGGGA
**TLK1_F2**	ATTACGCCTCGGGCACTTTA
**TLK1_R2**	TGGTAGAGGGTGCCTGAGAA

Supplementary Table 2. RT-PCR primers for TLK1 and TLK2.

Gene	Chr	ER+% (n=704)	LumA% (n=224)	LumB% (n=127)
TLK2	chr17	10.5	7.6	21.3
AURKA	chr20	7.0	4.0	13.4
PLK1	chr16	4.0	8.0	3.1
AURKC	chr19	2.0	2.7	0.8
CDK4	chr12	2.0	1.3	6.3
ATR	chr3	0.6	0.0	0.8
BUB3	chr10	0.6	0.0	1.6
CDK6	chr7	0.4	0.0	1.6
CHEK2	chr22	0.4	0.4	0.0
PLK2	chr5	0.4	0.0	0.8
PLK3	chr1	0.1	0.0	0.0
BUB1B	chr15	0.1	0.4	0.0
CHEK1	chr11	0.1	0.0	0.8
AURKB	chr17	0.1	0.0	0.0
BUB1	chr2	0.0	0.0	0.0
ATM	chr11	0.0	0.4	0.0
TLK1	chr2	0.0	0.0	0.0

Supplementary Table 3. The frequency of genomic amplifications of known cell cycle checkpoint kinases in invasive breast cancer The frequencies of genomic amplifications of known cell cycle checkpoint kinases (Nat Rev Drug Discov, 2009, 8:547) in breast cancer were analyzed based on Affymetrix SNP 6.0 copy number data from TCGA.

Supplementary Reference

1. Sillje HH, Takahashi K, Tanaka K, Van Houwe G, Nigg EA. Mammalian homologues of the plant Tousled gene code for cell-cycle-regulated kinases with maximal activities linked to ongoing DNA replication. *EMBO J* **18**, 5691-5702 (1999).
